# Deciphering the Impact of Temperature on Pleiotropic Consequences of RNA Polymerase Mutations

**DOI:** 10.1093/molbev/msaf226

**Published:** 2025-09-16

**Authors:** Deniz Ozbilek, Jake K Soley, Danna R Gifford, Christopher G Knight, Simon C Lovell, Mato Lagator

**Affiliations:** Division of Evolution and Genomic Sciences, School of Biological Sciences, Faculty of Biology, Medicine and Health, University of Manchester, Manchester M13 9PL, UK; Division of Evolution and Genomic Sciences, School of Biological Sciences, Faculty of Biology, Medicine and Health, University of Manchester, Manchester M13 9PL, UK; Division of Evolution and Genomic Sciences, School of Biological Sciences, Faculty of Biology, Medicine and Health, University of Manchester, Manchester M13 9PL, UK; School of Natural Sciences, Faculty of Science and Engineering, University of Manchester, Manchester M13 9PL, UK; Division of Evolution and Genomic Sciences, School of Biological Sciences, Faculty of Biology, Medicine and Health, University of Manchester, Manchester M13 9PL, UK; Division of Evolution and Genomic Sciences, School of Biological Sciences, Faculty of Biology, Medicine and Health, University of Manchester, Manchester M13 9PL, UK

**Keywords:** temperature, RNA polymerase, genotype–phenotype–fitness mapping, pleiotropy, antibiotic resistance

## Abstract

Despite occurring in an essential molecule, mutations in RNA polymerase readily emerge and elicit complex pleiotropic effects across different levels of biological organization, which are all modulated by environment. We investigated the impact of temperature on the effects of six mutations on sequence, structure, transcriptome, and organismal traits. We found temperature altered the transcriptomic response and key organismal traits such as growth rate and biofilm formation in a genotype-specific manner. Critically, mechanistic insights into the possible drivers of mutational effects emerged only when examining the relationships between different levels of organization: location of mutations in the tertiary structure and distance to key interacting molecules partly explained the observed transcriptomic differences, which in turn drove the impact of mutations on organismal traits. While falling short of capturing the full complexity of the system, our findings underscore the benefits of integrating insights across multiple biological levels to understand the relationship between environment and mutational effects in molecules with extensive pleiotropic effects.

## Introduction

**Fig. 1.**
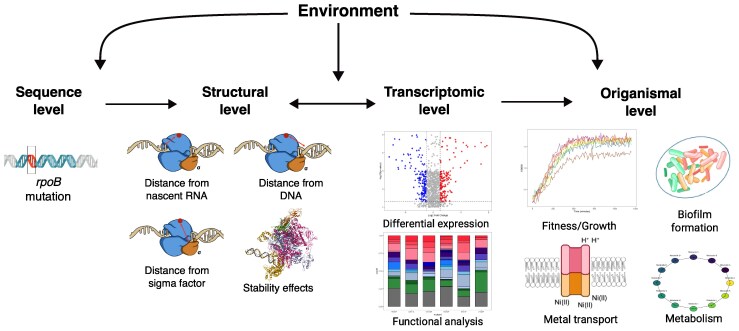
A holistic approach to understanding the effects of mutations with complex pleiotropic effects across different levels of biological organization. We adopt this approach to study some of the major consequences of *rpoB* mutations, specifically focusing on sequence, structural, transcriptomic, and organismal levels. Note that we include consequences both on an individual and on the population within the “organismal” level, for simplicity. This illustration captures some of the major, but not all possible, consequences of RNAP mutations. In particular, mutations in RNAP can alter its molecular function (transcription initiation, elongation, and/or termination)—potential consequences that were beyond the scope of our study.

RNA polymerase (RNAP) is one of the most important and highly conserved molecules across all domains of life. And yet, when challenged with antibiotics that target it, such as rifampicin, mutations in RNAP emerge with surprising frequency (Goldstein 2014; Alifano et al. 2015). In addition to providing resistance, these mutations also have strong pleiotropic effects across various levels of biological organization (Fig. 1; Qi et al. 2014; Bershtein et al. 2017; Trauner et al. 2021). As such, this seemingly simple system where bacteria acquire a single point mutation in RNAP, in reality elicits an enormously complex set of responses, altering molecular structure and function, which scale up to influence cellular processes and organismal fitness. This complexity of downstream consequences to point mutations limits our ability to understand and predict the evolution of one of the most essential molecules in biology, making RNAP an ideal study system to refine approaches to genotype–phenotype–fitness mapping.

At the sequence level, nonsynonymous point mutations in the *rpoB* gene give rise to rifampicin resistance via alterations to the structure of the binding site that reduce rifampicin binding (Fig. 1). The second level of biological organization affected by mutations in a subunit of RNAP is the structural, whereby mutations alter protein structure and stability. The consequences of changing the structure of RNAP may include alterations to the interactions with key partners—other subunits of RNAP, DNA, RNA, and σ factors. Changes to the structure and/or interactions with other key molecules can affect core functions of RNAP—initiation, elongation, and termination of transcription (Portelli et al. 2018; Vedithi et al. 2018). The mutational effects on the structural and molecular levels interact to induce genome-wide transcriptional dysregulation of a large number of genes (De Knegt et al. 2013; Xiao et al. 2017; Soley et al. 2023). Dysregulated gene expression as a consequence of RNAP mutations can interfere with various biological processes, such as the production of antibiotics and metabolites (Tanaka et al. 2013), decreased cell motility (Cutugno et al. 2020), salt tolerance (Cutugno et al. 2020), and host immune responses (Bobba et al. 2023). In doing so, dysregulation of expression affects organismal functions and fitness (Fig. 1; Bershtein et al. 2015; Utrilla et al. 2016). Although many of the consequences of *rpoB* mutations have been previously studied, they are typically studied independently of each other. As such, we lack the understanding of how changes at one level of biological organization affect changes at other levels, preventing predictions of mutational effects and their consequences for evolution.

Understanding how mutations in RNAP affect different levels of biological organization (Fig. 1) is further complicated by the potential environmental dependence of *rpoB* mutation effects at each level (Song et al. 2014; Gifford et al. 2016; Hinz et al. 2024). For example, mutations conferring low fitness in one environment can be neutral in another and vice versa (Soley et al. 2023). Although previous works explored how the fitness of *rpoB* mutants is altered in response to environmental fluctuations (Yubero and Poyatos 2021; Choudhury et al. 2023), the mechanisms underlying these responses remain poorly understood. Especially understudied are the consequences of environmental change on the effects of RNAP mutations across different levels of biological organization. We might, for example, expect mutations that are more structurally disruptive to also be more disruptive to transcription and consequently to fitness. We might also expect that environmental stressors, such as heat, would exacerbate such effects.

Here, we explore how one important environmental variable, temperature, affects the consequences of six *rpoB* mutations in *Escherichia coli*. We characterize mutational effects at several different levels of biological organization at two different temperatures: 37 °C, the optimal growth temperature for *E. coli*; and 42 °C, to understand how modest heat stress influences the effects of *rpoB* mutations. Our aim was not only to characterize the consequences of these mutations within each level of biological organization but also to find explanatory links between different levels. In doing so, we also provide a template on how to study biological systems with complex, pleiotropic effects to allow for their better understanding and to render their evolution more predictable: by dissecting mechanistically mutational effects across different levels of biological organization and connecting how changes at one level relate to changes at other levels. Our findings, which form an early attempt to explain the consequences of mutations across multiple layers of a highly pleiotropic system, highlight the value of multilevel frameworks (Bershtein et al. 2015; Choudhury et al. 2023; Grah et al. 2025) for understanding mutational effects and provide a foundation for predicting evolutionary trajectories in a system with high functional interconnectivity.

## Results

**Fig. 2.**
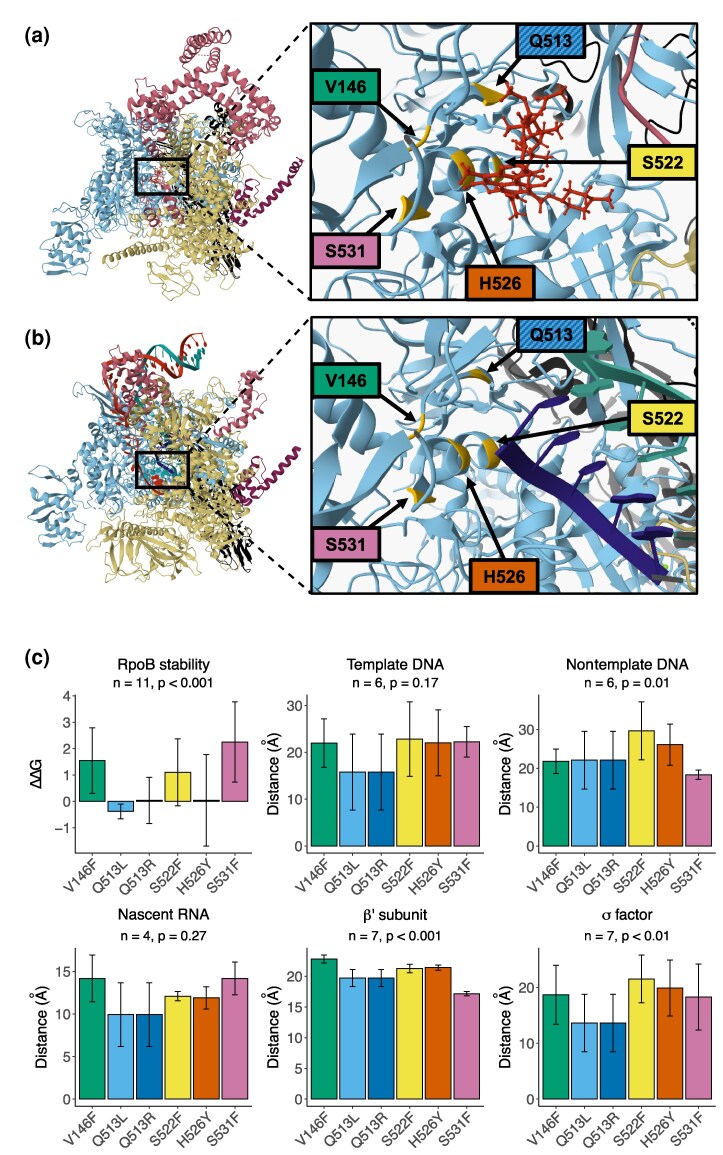
Effects of *rpoB* mutations on structural level. a) Proximity of mutated residues (yellow in zoomed image) in RpoB (cyan) to rifampicin (red) in RNAP quaternary structure (light yellow: β′ subunit, black: α subunit, purple: ω subunit, pink: σ factor; PDB ID: 4KMU). b) Same as a) but showing proximity to DNA and RNA during transcription (dark blue: nascent RNA, teal: template DNA strand, red: nontemplate DNA strand; PDB ID: 6UU9). c) Distances between mutations and interacting partners critical for transcription; and the predicted impact of mutations on RNAP stability (bottom right—note that positive *ΔΔ*G values indicate destabilization of the protein and negative values are stabilizing). Data are means of predictions from different PDB structures (see Table S1 and Materials and Methods); error bars represent standard deviation. Colors correspond to mutant labels in a/b.

To investigate how temperature influences the effects of *rpoB* mutations, we selected six mutants each with a single point mutation in *rpoB* conferring resistance to rifampicin: V146F, Q513L, Q513R, S522F, H526Y, and S531F (Fig. 2a and b). Strains were generated with a fluctuation assay-based approach, and the presence of a single mutation was confirmed with whole-genome sequencing, as previously described (Soley et al. 2023). Genomes of the six strains were otherwise identical to each other and that of the wild type.

### Structural Effects of RNAP Mutations

Protein stability, protein–protein interactions, and protein–ligand interactions are some of the most dominant biophysical traits driving the structural effects of mutations in RNAP (Bershtein et al. 2017). To function properly, the β subunit (RpoB) must fold in reasonable time, remain folded, and stably interact with other RNAP subunits, σ factors, and nucleic acids. To study the consequences of six *rpoB* mutations at the structural level, we computationally predicted how each mutation affected the structural stability of RNAP by calculating *ΔΔ*G for 11 crystal structures capturing different RNAP conformations (Table S1). We calculated *ΔΔ*G by comparing the change in Gibbs free energy between the unfolded and folded state of the wild-type protein with the equivalent change in energy of the mutated protein. We found that *ΔΔ*G varied significantly between mutants (F_5,24_ = 9.49, *P* < 0.001): three amino acid substitutions (V146F, S522F, and S531F) destabilize RpoB, while two (Q513L and H526Y) were stabilizing (Fig. 2c). Q513R was predicted to have a negligible impact on protein stability, with a *ΔΔ*G very close to 0.

The stability of RNAP is unlikely to be the only important alteration at the structural level. For example, conformational changes in protein folding may alter interactions with other molecules, affecting gene transcription. To explore the potential impact of mutations on the functioning of RNAP indirectly, we measured the Euclidean distance between mutated residues and the regions critical for transcription as well as key interacting molecules. Specifically, we calculated the shortest distance between each mutation (which sit within the RNA exit channel, at the site of rifampicin binding—Fig. 2a and b) and β′ subunit (RpoC), different σ factors, nascent RNA, and DNA (both template and nontemplate strand), in several different Protein Data Bank (PDB) structures of RNAP (Table S1). We found no significant difference in the distance to template DNA, or to RNA, between mutants (F_5,30_ = 1.66, *P* = 0.17 [template DNA]; F_5,12_ = 1.48, *P* = 0.27 [RNA]), suggesting that any mutation-specific effects at higher levels of organization are unlikely to be a result of these interactions (Fig. 2c). In contrast, the distance between the mutated residues and the nontemplate strand of DNA, β′, or σ factors differed significantly between *rpoB* mutants (F_5,30_ = 3.64, *P* = 0.01 [nontemplate DNA]; F_5,36_ = 34.14, *P* < 0.001 [β′]; F_5,36_ = 3.94, *P* < 0.01 [σ factor]; Fig. 2c). We hypothesize that mutations closer to these key sites have the potential to exert a stronger effect on RNAP function and therefore have a greater influence on fitness—a hypothesis we will test in relation to transcriptomic- and organismal-level effects of *rpoB* mutations in subsequent sections.

To explore if temperature alters the structural-level consequences of *rpoB* mutations, we modeled the impact of temperature on protein stability using FoldX “temperature” parameter, applied to all RNAP structures used in this study. Changes to temperature from 37 to 42 °C did not alter protein stability substantially in any of the mutants, suggesting some other mechanism by which temperature influences the pleiotropic effects of *rpoB* mutations (Fig. S1). An important caveat to this analysis is that computational tools for predicting the effects of mutations on protein stability are not trained on datasets for which the effects of mutations are measured at different temperatures (Schymkowitz et al. 2005). Furthermore, existing computational tools do not predict changes to distances between regions in a protein as a function of temperature, preventing us from modeling the impact of temperature on the distance between mutations and various regions critical for transcription. However, previous measurements of thermal expansion in proteins suggest that changing temperature from 37 to 42 °C would increase distance approximately isotropically and by around 0.075% (Frauenfelder et al. 1987; Tilton et al. 1992)—a change that is much lower than the coordinate error in the available RNAP structures (Cruickshank 1999). Therefore, as we investigate the link between the structural and the transcriptomic/organismal consequences of *rpoB* mutations, we will conservatively assume that the rank order of stability we identified among mutants (Fig. 2c) is maintained across temperatures.

### Transcriptomic Effects of RNAP Mutations

**Fig. 3.**
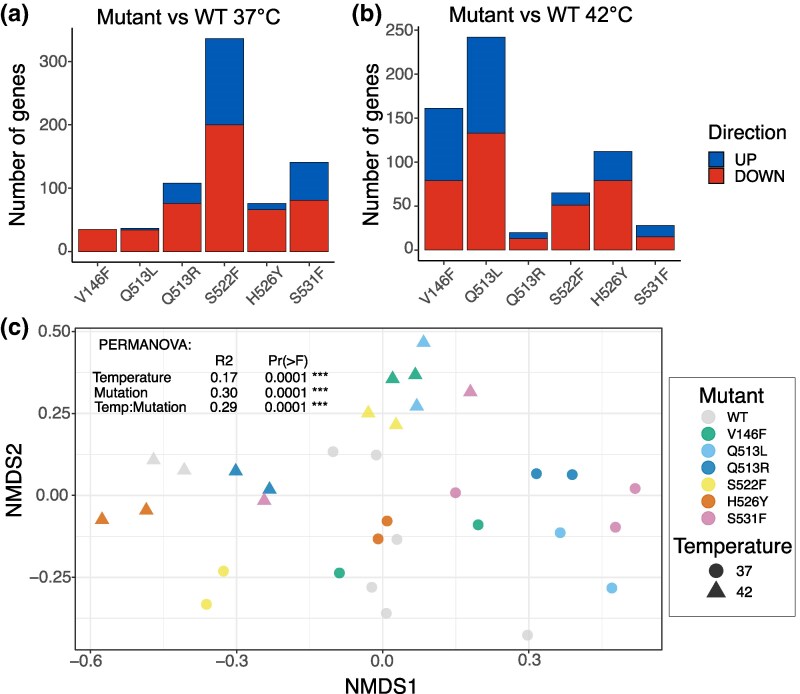
Effect of *rpoB* mutations on the transcriptomic level. a) Number of DEGs in each *rpoB* mutant grown at 37 °C or b) 42 °C. c) NMDS plot of normalized RNA-seq results of six mutants and the WT at two temperatures. Plot made using normalized RNA-seq count data matrix. For the complete model output, see Table S2.

To investigate how temperature impacts the effects of *rpoB* mutations at the transcriptomic level, we performed RNA-seq experiments at 37 and 42 °C to identify the transcriptomic-level effects of RNAP mutations. This allowed us to make several comparisons—the “mutation” effect when grown at 37 and 42 °C (i.e. differentially expressed genes—DEGs—in each mutant when compared with the wild type at each temperature), and the “temperature” effect of each mutant (e.g. DEGs of the same mutant grown at 42 versus 37 °C). As previously observed at 37 °C (Soley et al. 2023), the effect of mutations on the transcriptomic level was highly genotype-dependent at both temperatures, with the number of DEGs dependent on the mutation (Fig. 3a and b).

Through the analysis of the temperature effect on transcription we found that, although temperature played an important role, mutant identity was the key determinant of transcriptomic consequences of RNAP mutations in our study. There was almost twice as much genotype-specific variation (permutational multivariate analysis of variance [PERMANOVA]; *r*^2^ = 0.3, *P* < 0.001) and genotype-specific response to temperature (PERMANOVA; *r*^2^ = 0.29, *P* < 0.001) compared with temperature-specific variation (PERMANOVA; *r*^2^ = 0.17, *P* < 0.001; Figs. 3c and S2; Table S2).

**Fig. 4.**
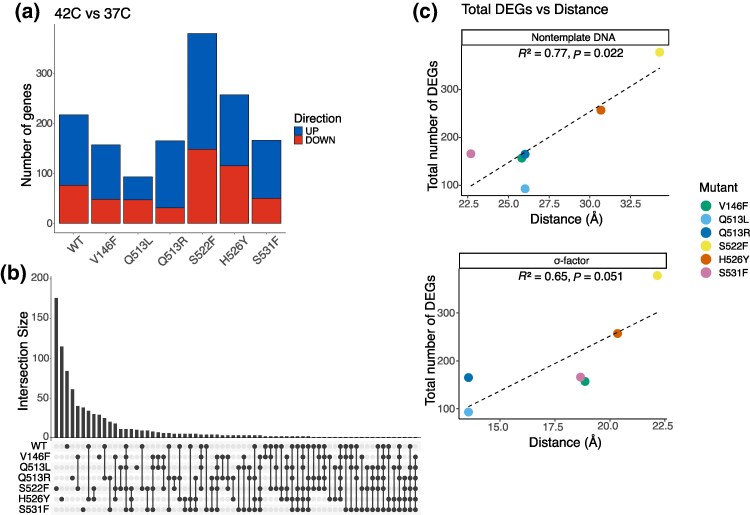
Impact of temperature on the transcriptomic consequences of *rpoB* mutations. a) Number of DEGs in each genotype when grown at 42 °C versus 37 °C. b) UpSet plot showing the number of unique and overlapping DEGs. Intersections with less than five genes removed to improve legibility. c) Correlations between total number of DEGs/number of downregulated genes and the distance between each mutated residue and the nontemplate strand of DNA. Mean distances across all structures modeled are shown; for correlations within each individual structure, see Table S4.

Our results point to an inconsistent effect of temperature on the expression profiles of mutants, as the number of DEGs at 42 versus 37 °C was dependent on the mutation: the total number of DEGs ranged from 93 (Q513L) to 378 (S522F; Fig. 4a). In all mutants except Q513L, more genes were upregulated than downregulated at 42 versus 37 °C (t(6) = 4.40, *P* < 0.005; Fig. 4a). While temperature led to dysregulation of a large number of genes across all six mutants, the majority of genes were exclusively differentially expressed in only a single mutant (328/567 upregulated genes; 342/401 downregulated genes). In other words, the identity of the dysregulated genes was strongly mutation-dependent, with even the two substitutions at the same residue (Q513) sharing only 17 DEGs (Fig. 4b). Critically, while all mutant pairs shared some DEGs (Table S3), no genes were differentially expressed across all mutants (Fig. 4b). This is surprising because we expected a portion of the DEGs to be shared across all mutants and the wild type as a generic response to temperature change (Li et al. 2018). Instead, our data shows that the response to temperature change is dominated by the effects of *rpoB* mutations, which substantially alter the transcriptomic response to temperature in genotype-specific manner.

### Linking Structural to Transcriptomic Effects

Understanding the consequences of mutations in highly pleiotropic molecules requires not only examining how mutations alter function “within” each level of biological organization but also how the consequences at one level propagate to the next one. To explore potential links between the structural and the transcriptomic consequences of *rpoB* mutations, we looked for correlations between the changes at structural and transcriptomic levels of organization. Namely, we interrogated the relationship between structural stability, the distance between mutations and key interaction partners, and gene expression changes. We found no significant structural-to-transcriptomic relationships either at 37 or 42 °C independently (Table S4), suggesting that the effects of mutations on transcriptomes at a given temperature were not driven by any of the structural-level changes we measured.

In contrast, the transcriptomic response of mutants (ie comparing the number of DEGs of each mutant at 42 versus 37 °C) was positively correlated with two structural-level factors in several structures we modeled: the distance between the mutated residue and the nontemplate DNA strand and the distance to the σ factor (Fig. 4c). These distances had a greater effect on downregulation, as the number of downregulated genes correlated significantly with the distance to nontemplate DNA and the σ factor, unlike the number of upregulated genes (Table S4; Fig. S3). The small scale of our dataset prevented us from meaningfully dissecting how amino acid properties and residue position contribute to differential gene expression. We did not identify a significant relationship between the number of DEGs (neither the total number nor the number of upregulated/downregulated) and the distance between the mutation and the β′ subunit. Interestingly, we found no correlation between the stability of RpoB (average *ΔΔ*G across all structures used) and the total number of DEGs (adjusted *r*^2^ = 0.0289, *P* = 0.3442). Put together, our data point to a key, and positive, interaction between the distance of a mutation to σ factors and DNA, suggesting that *rpoB* mutations directly modulate the effect of temperature on the interaction between RpoB, DNA and σ factors.

### Linking Transcriptomic to Organismal Effects

**Fig. 5.**
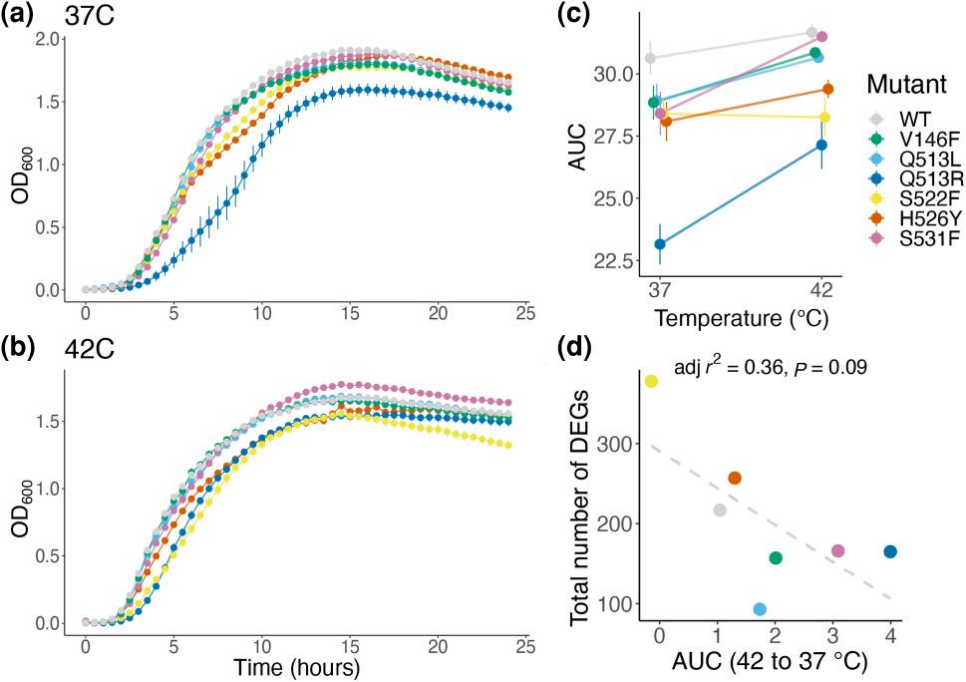
Growth curve fitness of RNAP mutants and the wild type at two temperatures. a) Growth curves at 37 °C and b) 42 °C. c) Scatter plot showing AUC of each mutant and the wild type at both temperatures. Data are mean of 12 biological replicates; error bars are standard error of the mean. d) Correlations between the total DEGs at 42 versus 37 °C and the AUC difference between temperatures. Gray line shows results of linear regression analysis.

One of the potential consequences of RNAP mutation-induced transcriptomic dysregulation at the organismal level is on bacterial fitness, which we measured as area under the curve (AUC) of mutant growth curves (Fig. 1). *RpoB* mutants exhibited growth differences within each temperature, with Q513R showing markedly less growth at both 37 and 42 °C (Fig. 5). When considering growth between the two temperatures, for the majority of *rpoB* mutants and the wild type, there was a slight but not significant increase in fitness when grown at 42 °C compared with 37 °C, while two mutants (Q513R and S531F) exhibited a larger and significant increase in growth at the higher temperature (Fig. 5c; Table S5). As such, increasing temperature decreased the growth deficit of Q513R compared with the wild-type and other mutants.

To investigate if transcriptomic-level effects drove the impact of temperature on mutant fitness, we correlated the number of DEGs (42 versus 37 °C) and the difference between AUCs of each mutant between the two temperatures and found a negative (but not significant) relationship (Fig. 5d). This suggests, albeit counterintuitively, that a mutation resulting in a higher number of DEGs may lead to a smaller difference in fitness between the two temperatures. As such, the consequences of mutations on bacterial growth are driven more by the identity of specific dysregulated genes rather than the total number of dysregulated genes.

**Fig. 6.**
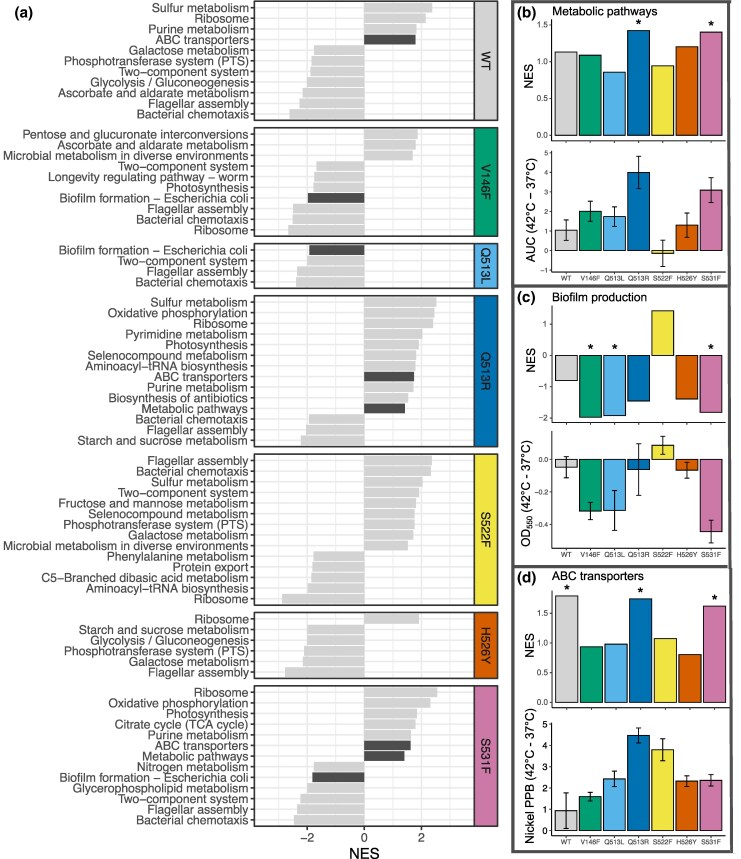
Relating transcriptomic-level changes to organismal responses to temperature. a) GSEA of DEGs for each genotype at 42 versus 37 °C, showing all KEGG categories with significant normalized enrichment scores. Bars highlighted in dark gray are focal categories for experimental analysis [which are shown again in b), c), and d); highlighted with arrows]. b) Top: NES for “Metabolic pathways” KEGG group for each genotype. Bottom: Difference in AUC from growth curves of each genotype at 42 °C minus 37 °C (*n* = 12). c) Top: NES for “Biofilm formation” KEGG group for each genotype. Bottom: Difference in level of biofilm (measured as optical density at 550 nm—see Materials and Methods) at 42 °C minus 37 °C (*n* = 6). d) Top: NES for “ABC transporters” KEGG group for each genotype. Bottom: Difference in cellular nickel PPB at 42 °C minus 37 °C (*n* = 3). Error bars in b), c), and d) show error propagation (standard error of the mean).

However, this approach based on looking only at the total number of DEGs fails to consider their function. To contextualize genes with regard to their function, we performed a functional analysis of transcriptomic data using Gene Set Enrichment Analysis (GSEA). GSEA ranks genes (here by log-fold change in expression) before testing for enrichment of gene sets. We used the Kyoto Encyclopedia of Genes and Genomes (KEGG) database for high-level functional classification of genes (Fig. 6a). The same two mutants whose fitness (as AUC) was significantly higher at 42 °C compared with 37 °C (Q513R and S531F) were also the only ones with a significant overexpression of genes in metabolic pathways at 42 °C (Figs. 6b and S4a). While many factors might impact bacterial growth, overexpression of metabolic genes is certainly one of them (Cassey et al. 1998; Anand et al. 2021; Rabbers and Bruggeman 2022), lending credence to the observed correlation. For instance, both Q513R and S531F were found to overexpress genes involved in the production of ATP-synthase (*atpA/C/G/D*)—a ubiquitously expressed enzyme shown to maximize growth rate (Rabbers and Bruggeman 2022). The same two mutants also overexpressed genes that are a part of the pyruvate dehydrogenase complex (*aceE/F*) as well as genes involved in phosphorylation of pyruvate (*ppsA*) and conversion of L-lactate to pyruvate (*lldD*). Pyruvate is essential for aerobic growth and has been shown to increase the rate of aerobic respiration (Rabbers and Bruggeman 2022).

In addition to metabolic genes, we found altered expression of several other gene sets that could impact organismal function (Fig. 6a). We experimentally tested if differential expression of two functional categories that separated the mutants based on their response to temperature (biofilm formation and ATP synthase-binding casette (ABC) transporters) had measurable consequences at the organismal level. The only mutant with a modest increase in biofilm formation at 42 °C (S522F) was also the only mutant with the positive enrichment score for genes involved in biofilm formation (although neither normalised enrichment score (NES) nor biofilm formation data were statistically significantly different from wild type for this mutant). Genes involved in biofilm formation were significantly enriched among downregulated genes in V146F, Q513L, and S531F, suggesting biofilm formation is suppressed when those mutants are grown at 42 °C compared with at 37 °C (Fig. 6a). Correspondingly, the experimentally measured biofilm levels varied significantly between mutants (F_6,77_ = 3.21, *P* = 0.007), with the same three mutants showing reduced biofilm levels at 42 versus 37 °C (Figs. 6c and S4b).

In contrast to metabolism and biofilm formation, we did not identify a linear link between ABC transporter activity and DEG set. To measure the activity of ABC transporters, which were enriched in the response of only some of the mutants to temperature (Fig. 6a), we quantitatively assessed the concentration of a substrate metal that relies on the activity of ABC transporters—nickel (Eitinger and Mandrand-Berthelot 2000). While we found significant variation between the intracellular nickel concentrations of mutants (F_6,35_ = 7.38, *P* < 0.001), they did not correlate with differences observed at transcriptomic level (Figs. 6d and S4c). Although increased ABC transporter expression in Q513R at 42 versus 37 °C correlated with higher nickel levels, neither wild-type (WT) nor S531F showed similar correlations. Further, S522F had the second highest increase in intracellular nickel concentrations, where enrichment of ABC transporter genes was not seen among DEGs. The lack of correlation between the expression of ABC transporters and nickel concentrations between temperatures points to the complexity, and the difficulty, of relating mutational effects between different levels of biological organization.

Together, our results point to the functional identity of genes, rather than the total number of DEGs, that govern the relationship between transcriptional- and organismal-level responses to temperature. However, linking enriched functional categories to organismal phenotypes is sometimes linear (in the case of metabolism and biofilm formation) and sometimes more complex (ABC transporters).

### Connecting Across All Levels of Biological Organization

**Fig. 7.**
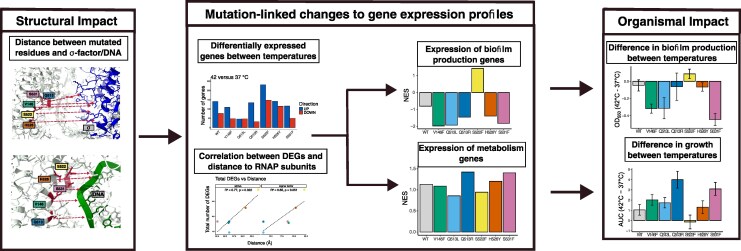
Connecting across all levels of biological organization. Summary of significant links between different levels of biological organization identified in this work.

In this work, we interrogated the relationship between adjacent levels of biological organization (Fig. 1), to identify if changes at one level correlated with changes at another. We identified several significant relationships that allow making a connection across all levels: the distance between mutated residues and σ factors/DNA affected how temperature modulated the number of DEGs, which it did in a function-specific manner resulting in predictable changes to organismal-level phenotypes (Fig. 7).

While the ability to draw any connection across all the investigated levels of biological organization in such a complex system is positively surprising, we do not think that our work presents a conclusive explanation of the forces that shape the consequences of *rpoB* mutations and how they are modulated by temperature. Instead, we present an early attempt to link mutational effects across different levels of biological organization, where both, the existence of linear connections and the lack thereof, elucidate mechanistic aspects of this complex system. For example, we identified a positive relationship between distance of a mutation to σ factor/DNA and the effect of temperature on the number of DEGs. This relationship suggests the possibility of thermal expansion playing a role in RNAP function and evolution—a relationship that has not been previously investigated. Conversely, the lack of a linear correlation between the number of DEGs and bacterial growth points to the poorly understood relationship between gene expression levels and organismal function.

## Discussion

Linking mutations to their consequences across various levels of biological organization remains particularly challenging for mutations with complex pleiotropic effects (Fig. 1). The problem is only further magnified as the mutational effects at any of the levels can be altered by the environment (Martin and Lenormand 2006; Des Marais et al. 2013; Saltz et al. 2018; Cummings and Kavlock 2004). In this study, we explored how a change in temperature modulates the consequences of single point mutations in RNAP across several levels of biological organization—structural, transcriptomic, and organismal. Effects of mutations on the molecular level were not studied because there is not an existing modeling framework to simulate the effects of mutations on initiation, elongation, and termination processes and the experiments required to do so are beyond the scope of this study. We identified genotype- and temperature-specific variations in the structural, transcriptomic, and organismal traits of *rpoB* mutants and their response to temperature change. Especially interesting was the impact of temperature on the transcriptome, which was heavily dependent on mutant identity (Fig. 4b).

A major novelty of our work lies in characterizing both, the links between different levels of biological organization and the difficulties in doing so, as both the presence and the absence of links point to the complexity of mechanistic underpinnings that drive the interaction between genotype and temperature. We revealed heterogeneity in how mutational effects at different levels of biological organization relate to each other, some mapped in a linear way while others exhibited nonlinear, unpredictable interactions. In attempting to link structural to transcriptomic effects, we found correlations between the number of DEGs of each mutant at the two temperatures and the distances to nontemplate DNA and to σ factors, suggesting a possible mechanism behind transcriptomic dysregulation (Fig. 4). Particularly surprising was the fact that the correlation was positive, suggesting a nonintuitive relationship where mutations that are further away from key interacting molecules have a larger transcriptional response to temperature change. Temperature-induced changes to transcriptome preferentially affected certain organismal functions, leading to measurable organismal-level consequences such as altered biofilm formation and metabolism (Fig. 6). Thus, we identified a through-line connecting all the investigated levels of organization (Fig. 7). However, there were cases, such as that of ABC transporters for nickel, where measured levels of intracellular nickel did not match the observed expression patterns (Fig. 6). Variation in the complexity and linearity of connections between different levels of biological organization points to the challenges in tracing how environmental, cellular, and molecular factors shape mutational effects.

Differences in the impact of temperature on the effects of RNAP mutations can have implications for the evolutionary potential of those strains. We found that two mutants exhibited higher growth at 42 °C, relative to 37 °C, purely as a consequence of their single point mutation in RNAP (Fig. 5c). This could provide an evolutionary advantage in environments where temperatures regularly vary, such as during an infection (Sköld-Chiriac et al. 2015; Solar Venero et al. 2024; Van Eldijk et al. 2024). More broadly, the distribution of different resistance alleles globally could be influenced by their ability to grow at different temperatures in diverse climates, potentially influencing which rifampicin resistance alleles spread in different regions. Differences in the effect of temperature on biofilm formation of different mutants suggest that adaptation to specific niches can depend on genotype-by-environment interactions (Fig. 6c). These adaptations would be missed by simply measuring bacterial growth rate, highlighting an advantage of teasing apart the complex phenotypic consequences of mutations.

We note several limitations with the present study that prevent comprehensively linking mutations to their consequences at all levels of biological organization (Fig. 1). Our assumption that the temperature change influences the structural effects of different mutations equally, so that the rank order is maintained in both temperature environments, may not be accurate. Previous work has highlighted the complex genotype-by-environment interactions that influence the consequences of *rpoB* mutations (Gifford et al. 2016; Soley et al. 2023). Such interactions may result in the structural effects of some mutations being more pronounced than others when the temperature is changed. Our assumption was necessary given the lack of computational tools to predicting temperature effects on molecular structures, with experimental investigations into the consequences of temperature on RNAP structure lacking. The approach we adopted (Fig. 1) will benefit from further studies identifying various potential consequences of RNAP mutations that we did not account for. Especially critical will be to understand how RNAP mutations and temperature alter the molecular functioning of RNAP—the initiation, elongation, and termination of transcription. Other phenotypes at all levels of organization can potentially be impacted by RNAP mutations (Jin and Gross 1989; Cui et al. 2010; Qi et al. 2014), with the lack of mechanistic models of those consequences limiting their integration into holistic analyses like ours. Finally, our focus on a relatively small number of mutations in *E. coli* puts limitations on the generalizability of our findings; however, the mutations we investigated are found in other species with similar effects on transcription (Vogwill et al. 2016; Trauner et al. 2021).

Despite a wealth of research on rifampicin-resistant mutants, predicting the complex downstream effects of RNAP mutations on cellular phenotypes and fitness remains a challenge. Here, we build on previous work identifying many of the effects of *rpoB* mutations (Jin and Gross 1989; Qi et al. 2014; Cutugno et al. 2020; Yang et al. 2023) to not only describe their consequences within individual levels of biological organization but also to identify how the consequences at one level propagate to other. This holistic approach contrasts with most studies, which focus on the consequences at one or two levels of organization. Adopting this approach identified structural and transcriptional consequences of RNAP mutations that are modulated by temperature, with unexpected relationships among organizational levels. We argue that accounting for the consequences of mutations across different levels of biological organization, and how those levels relate causally, is critical for understanding and predicting the complex pleiotropic consequences of mutations.

## Materials and Methods

### Bacterial Strains

All mutants used in this study were evolved from an ancestral WT strain—*E. coli* BW25113 *ΔtolC* from the Keio collection (Baba et al. 2006). The *tolC* deletion was used to ensure the selection of *rpoB* mutations and avoid resistance through efflux pump activity as many efflux mechanisms require TolC to function. *rpoB* mutants were selected with a fluctuation assay-based approach, and the presence of a single mutation was validated with whole genome sequencing (WGS) as described in Soley et al. (2023). From the 12 mutants we generated in this study, we selected 6 mutants to cover a variety in fitness costs and whether they occur both in the same and different residues of RpoB (tw2o mutants were to the same and the remaining 4 to different residues).

### Predictions of Structural-Level Effects

Protein structures used for computational calculations of structural-level effects were downloaded from the PDB (RCSB.org; Berman et al. 2000). The PDB accession ID of all structures used can be found in Table S1. To estimate Euclidean distance from each mutation to key functional sites within RNAP, PDB structures were imported to R with the *bio3d* package (Grant et al. 2006) and then distances were calculated from C-alpha of the mutated residue to every C-alpha on the relevant site or subunit (RpoC, σ factor, template/nontemplate DNA strands, nascent RNA strand). Distances were sorted, and the shortest distance for each site was used. Gibbs free energy of protein folding values (*ΔΔ*G) for mutated RpoB was predicted in FoldX (Schymkowitz et al. 2005) using the *BuildModel* command. Gibbs free energy of protein folding values (*ΔΔ*G) at different temperatures was predicted using the *BuildModel* command with the “temperature” parameter set to different temperatures using PDB structures listed in Table S1 (Fig. S1). A one-way analysis of variance (ANOVA) was performed to compare the effect of different *rpoB* mutations on each structural characteristic.

When calculating correlations between structural and transcriptomic effects, we used PDB structure 6UU9 for distances to RNA as it had the longest RNA chain; distances to RpoC were predicted from the same structure as there was little variation between structures (Fig. 2). Distances to σ factor and both template and nontemplate DNA strands were used from multiple PDB structures and tested separately for correlations, as these distances varied between structures (Fig. 2). When testing correlations with *ΔΔ*G, the mean *ΔΔ*G of all structures was used. Correlations were calculated in R with the *lm* command, using default settings. We used multiple PDB structures to capture a range of structural conformations of RNAP bound to different σ-factors and different stages of transcription (Table S1).

### Measurement of Transcriptomic Effects

We performed RNA-seq analysis on six *rpoB* mutants and the wild type at two temperatures (37 and 42 °C). RNA was extracted as in Soley et al. (2023), and then samples were sent to “Azenta Life Sciences” (Germany) for quality control, rRNA depletion, library preparation, and sequencing with the Standard RNA-Seq service (Illumina NovaSeq, 150 bp paired end reads). Raw sequencing reads were trimmed with *Trim Galore* (*v0.6.7*; https://github.com/FelixKrueger/TrimGalore) using default parameters to remove sequencing adapters and low-quality regions. Reads shorter than 50 bp after trimming were then discarded. Any remaining rRNA reads were removed with *BBDuk* (*v38.18*; https://sourceforge.net/projects/bbmap) using the bundled rRNA k-mers database. Trimmed mRNA reads were aligned to the reference genome (CP009273) using *Bowtie2* (*v2.2.5*; Langmead and Salzberg 2012), and then transcript count tables were constructed using *featureCounts* (*v2.0.1*; Liao et al. 2014). Untransformed counts were adjusted for batch effects based on negative binomial regression with the *ComBat-seq* tool available in R/Bioconductor package *sva* (*v3.48.0*; Zhang et al. 2020). Differential expression analysis was performed with *DESeq2* (*v1.40.1*; Love et al. 2014) to all genes with rowSums ≥10 in the adjusted count table. We constructed the *DESeq2* model to account for temperature and mutant as main terms, as well as an interaction term between them. When conducting comparisons between differential expression of a given mutant at two temperatures, we releveled that mutant at 37 °C to the reference level within DESeq model before extracting the temperature effect, which allowed us to look at genotype-specific responses to temperature (for full details of the model, see Supplementary material, Source Code 1). Significantly DEGs were defined as genes with a log-fold change >1 at an adjusted *P*-value for false discovery rate <0.05. Nonmetric multidimensional scaling (NMDS) and a PERMANOVA model were performed to analyze the impact of temperature and genotype on differential gene expression. Distance matrix for NMDS plot in Fig. 3c was created using *vegdist* function with Bray–Curtis distance available in R/vegan package. PERMANOVA model was performed on the same distance matrix using the *adonis2* available in R/vegan package using the formula distance_matrix ∼ temperature × mutation + experiment + replicate, permutations = 9,999. principal component analysis (PCA) plot in Fig. S2 was created using the *plotPCA* function of the R/DESeq2 package. UpSet plots in Fig. 4 were created with *UpSetR* (Lex et al. 2014; Conway et al. 2017) with intersections with five or more genes. Functional analysis of DEGs was performed using GSEA with KEGG terms (KEGG; Kanehisa et al. 2023), using the R/Bioconductor package *clusterProfiler* (*v4.8.2*; Wu et al. 2021).

### Correlations Between the Effects at Structural and Transcriptomic Levels

All linear models were run using the default settings of the *lm* function in R, with the formula *Var1∼Var2, method*  *=* “*qr*,” *where* Var1 and Var2 refer to variable1 and variabe2 in the Table S4, respectively; *qr* method refers to ordinary least squares. Correlations with adjusted *r*^2^ values >0.3 and false discovery rate (FDR)corrected *P*-value <0.05 were considered significant. All reported correlations are between the number of DEGs (separately for total, up, and downregulated number of DEGs) and distance to key interaction partners.

### Measurements of Organismal-Level Effects

#### Growth Curves

Growth curves were obtained using a CLARIOstar Plus microplate reader. Strains were grown overnight at 37 °C in lysogeny broth (LB) inoculated from frozen stocks and then diluted 1:1,000 into fresh media in microtiter plates. Plates were incubated in the microplate reader for 24 h with shaking, either at 37 °C or at 42 °C, and OD_600_ was measured every 10 min. Twelve biological replicates were taken for each mutant in each condition. Growth curves were analyzed in R with the package *growthcurver* (Sprouffske and Wagner 2016) to calculate the empirical AUC between 0 and 24 h. In subsequent analyses, AUC is used as the metric for bacterial fitness, as it most comprehensively reflects the underlying growth data.

#### Biofilm Formation

Strains were grown overnight in LB media and then diluted the following morning 1:100 into fresh media. One hundred microliters of diluted culture was added per well into 96-well plates, one for growth at 37 °C and one for growth at 42 °C, with 6 replicates per sample. Plates were incubated at the relevant temperature for 4 h without shaking. After incubation, plates were tipped to remove cells, then washed gently twice by gentle submersion in water. One hundred twenty-five microliters of a 0.1% crystal violet solution was added to each well, before incubation at room temperature for 15 min. Plates were then washed four times with water as before and then dried upside down overnight. To solubilize the crystal violet, 125 µL of 30% acetic acid was added to each well, and plates were incubated at room temperature for 15 min. Solubilized crystal violet was transferred to a new flat bottom microtiter plate, and then absorbance at 550 nm was quantified in a CLARIOstar Plus microplate reader.

#### ABC Transporter Activity

Strains were grown overnight at 37 °C in LB and then diluted the following morning 1:100 into 20 mL fresh media and grown to stationary phase at either 37 or 42 °C with shaking (200 RPM), with 3 biological replicates per sample. Two microliters of culture were taken for serial dilution. The remaining culture was centrifuged (2696 x *g*, 40 min) and pellets were resuspended in 10 mL Tris-EDTA (TE) buffer and then repeated for a total of 3 washes. After final wash, pellets were resuspended in 10 mL mQH_2_O and spun again (3,660 RPM, 10 min), and supernatant was removed before drying pellets in 80 °C oven overnight (without lids on Falcon tubes). The following morning, 0.5 mL 70% nitric acid was added to each tube, lids were replaced with parafilm to reduce evaporation, and samples were incubated overnight at 37 °C with shaking (200 RPM) to dissolve pellets. On the final day, samples were diluted in a final volume of 10 mL mQH_2_O and filter sterilized and then sent for analysis by Inductively Coupled Plasma Mass Spectrometry at the Manchester Analytical Geochemistry Unit for detection of nickel parts per billion (ppb).

For each organismal-level effect, the propagation of error was calculated for the difference between 42 and 37 °C (*σ_f_*), where *σ* is standard deviation:


$$\sigma_{f}=\;\sqrt{{\sigma^{2}}_{37^{\circ }C}+\;{\sigma^{2}}_{42^{\circ }C}}$$

One-way ANOVA was then performed to test the significance between different mutants (Fig. 6b to d; bottom plots).

## Supplementary Material

msaf226_Supplementary_Data

## Data Availability

All data underpinning this paper are provided as Supplementary material. All raw RNA-seq data have been deposited to NCBI under BioProject accession PRJNA1167737.
