# Supplementary material for: Deciphering the Impact of Temperature on Pleiotropic Consequences of RNA Polymerase Mutations
Source: Mol Biol Evol. 2025 Sep 16;42(10):msaf226. doi: 10.1093/molbev/msaf226 (PMC12503110; doi:10.1093/molbev/msaf226)
Supplement: msaf226_Supplementary_Data [file msaf226_supplementary_data.zip › Source_code1.html]

DEseq core analysis explanation


# DEseq core analysis explanation

#### Jake Soley

#### 2025-07-30

## Install and load packages

```
library('tidytext')
```

```
## Warning: package 'tidytext' was built under R version 4.3.3
```

```
library('DESeq2')
```

```
## Loading required package: S4Vectors
```

```
## Loading required package: stats4
```

```
## Loading required package: BiocGenerics
```

```
## 
## Attaching package: 'BiocGenerics'
```

```
## The following objects are masked from 'package:stats':
## 
##     IQR, mad, sd, var, xtabs
```

```
## The following objects are masked from 'package:base':
## 
##     anyDuplicated, aperm, append, as.data.frame, basename, cbind,
##     colnames, dirname, do.call, duplicated, eval, evalq, Filter, Find,
##     get, grep, grepl, intersect, is.unsorted, lapply, Map, mapply,
##     match, mget, order, paste, pmax, pmax.int, pmin, pmin.int,
##     Position, rank, rbind, Reduce, rownames, sapply, setdiff, sort,
##     table, tapply, union, unique, unsplit, which.max, which.min
```

```
## 
## Attaching package: 'S4Vectors'
```

```
## The following object is masked from 'package:utils':
## 
##     findMatches
```

```
## The following objects are masked from 'package:base':
## 
##     expand.grid, I, unname
```

```
## Loading required package: IRanges
```

```
## 
## Attaching package: 'IRanges'
```

```
## The following object is masked from 'package:grDevices':
## 
##     windows
```

```
## Loading required package: GenomicRanges
```

```
## Loading required package: GenomeInfoDb
```

```
## Loading required package: SummarizedExperiment
```

```
## Loading required package: MatrixGenerics
```

```
## Loading required package: matrixStats
```

```
## 
## Attaching package: 'MatrixGenerics'
```

```
## The following objects are masked from 'package:matrixStats':
## 
##     colAlls, colAnyNAs, colAnys, colAvgsPerRowSet, colCollapse,
##     colCounts, colCummaxs, colCummins, colCumprods, colCumsums,
##     colDiffs, colIQRDiffs, colIQRs, colLogSumExps, colMadDiffs,
##     colMads, colMaxs, colMeans2, colMedians, colMins, colOrderStats,
##     colProds, colQuantiles, colRanges, colRanks, colSdDiffs, colSds,
##     colSums2, colTabulates, colVarDiffs, colVars, colWeightedMads,
##     colWeightedMeans, colWeightedMedians, colWeightedSds,
##     colWeightedVars, rowAlls, rowAnyNAs, rowAnys, rowAvgsPerColSet,
##     rowCollapse, rowCounts, rowCummaxs, rowCummins, rowCumprods,
##     rowCumsums, rowDiffs, rowIQRDiffs, rowIQRs, rowLogSumExps,
##     rowMadDiffs, rowMads, rowMaxs, rowMeans2, rowMedians, rowMins,
##     rowOrderStats, rowProds, rowQuantiles, rowRanges, rowRanks,
##     rowSdDiffs, rowSds, rowSums2, rowTabulates, rowVarDiffs, rowVars,
##     rowWeightedMads, rowWeightedMeans, rowWeightedMedians,
##     rowWeightedSds, rowWeightedVars
```

```
## Loading required package: Biobase
```

```
## Welcome to Bioconductor
## 
##     Vignettes contain introductory material; view with
##     'browseVignettes()'. To cite Bioconductor, see
##     'citation("Biobase")', and for packages 'citation("pkgname")'.
```

```
## 
## Attaching package: 'Biobase'
```

```
## The following object is masked from 'package:MatrixGenerics':
## 
##     rowMedians
```

```
## The following objects are masked from 'package:matrixStats':
## 
##     anyMissing, rowMedians
```

```
library('gplots')
```

```
## 
## Attaching package: 'gplots'
```

```
## The following object is masked from 'package:IRanges':
## 
##     space
```

```
## The following object is masked from 'package:S4Vectors':
## 
##     space
```

```
## The following object is masked from 'package:stats':
## 
##     lowess
```

```
library('tidyverse')
```

```
## ── Attaching core tidyverse packages ──────────────────────── tidyverse 2.0.0 ──
## ✔ dplyr     1.1.2     ✔ readr     2.1.4
## ✔ forcats   1.0.0     ✔ stringr   1.5.0
## ✔ ggplot2   3.4.3     ✔ tibble    3.2.1
## ✔ lubridate 1.9.2     ✔ tidyr     1.3.0
## ✔ purrr     1.0.1
```

```
## ── Conflicts ────────────────────────────────────────── tidyverse_conflicts() ──
## ✖ lubridate::%within%() masks IRanges::%within%()
## ✖ dplyr::collapse()     masks IRanges::collapse()
## ✖ dplyr::combine()      masks Biobase::combine(), BiocGenerics::combine()
## ✖ dplyr::count()        masks matrixStats::count()
## ✖ dplyr::desc()         masks IRanges::desc()
## ✖ tidyr::expand()       masks S4Vectors::expand()
## ✖ dplyr::filter()       masks stats::filter()
## ✖ dplyr::first()        masks S4Vectors::first()
## ✖ dplyr::lag()          masks stats::lag()
## ✖ ggplot2::Position()   masks BiocGenerics::Position(), base::Position()
## ✖ purrr::reduce()       masks GenomicRanges::reduce(), IRanges::reduce()
## ✖ dplyr::rename()       masks S4Vectors::rename()
## ✖ lubridate::second()   masks S4Vectors::second()
## ✖ lubridate::second<-() masks S4Vectors::second<-()
## ✖ dplyr::slice()        masks IRanges::slice()
## ℹ Use the conflicted package (<http://conflicted.r-lib.org/ [conflicted.r-lib.org]>) to force all conflicts to become errors
```

```
library('pheatmap')
library('RColorBrewer')
library('genefilter')
```

```
## 
## Attaching package: 'genefilter'
## 
## The following object is masked from 'package:readr':
## 
##     spec
## 
## The following objects are masked from 'package:MatrixGenerics':
## 
##     rowSds, rowVars
## 
## The following objects are masked from 'package:matrixStats':
## 
##     rowSds, rowVars
```

```
library('ggpubr')
library('gridGraphics')
```

```
## Loading required package: grid
```

```
library('apeglm')
library('patchwork')
```

```
## 
## Attaching package: 'patchwork'
## 
## The following object is masked from 'package:genefilter':
## 
##     area
```

```
library('tidyr')
```

## Load featureCounts table and format sample table

```
# Read count data from file 
count_table <- read.table("ComBatSeq_adjusted_count_table.tsv", sep="\t", header=TRUE, row.names=1)

# Specify design variables
temperature <- factor(c(rep("37C", 16), rep("42C", 14)))
mutant <- factor(c("S522F", rep("H526Y",2), "S522F", rep("V146F",2), rep("Q513L",2), rep("Q513R",2), rep("S531F",2), rep("WT", 6), rep("H526Y",2), rep("Q513L",2), rep("Q513R",2), rep("S522F",2), rep("S531F",2), rep("V146F",2)))

# Create table listing samples; will be fed into the DESeq model for column data
sample_table <- data.frame(temperature = as.factor(temperature),
                           mutant = as.factor(mutant))

rownames(sample_table) <- colnames(count_table)

sample_table
```

```
##               temperature mutant
## J.37C.S522F.1         37C  S522F
## J.37C.H526Y.1         37C  H526Y
## J.37C.H526Y.2         37C  H526Y
## J.37C.S522F.2         37C  S522F
## J.37C.V146F.1         37C  V146F
## J.37C.V146F.2         37C  V146F
## J.37C.Q513L.1         37C  Q513L
## J.37C.Q513L.2         37C  Q513L
## J.37C.Q513R.1         37C  Q513R
## J.37C.Q513R.2         37C  Q513R
## J.37C.S531F.1         37C  S531F
## J.37C.S531F.2         37C  S531F
## J.37C.WT.3            37C     WT
## J.37C.WT.4            37C     WT
## D.37C.WT.1            37C     WT
## D.37C.WT.2            37C     WT
## D.42C.WT.1            42C     WT
## D.42C.WT.2            42C     WT
## D.42C.H526Y.1         42C  H526Y
## D.42C.H526Y.2         42C  H526Y
## D.42C.Q513L.1         42C  Q513L
## D.42C.Q513L.2         42C  Q513L
## D.42C.Q513R.1         42C  Q513R
## D.42C.Q513R.2         42C  Q513R
## D.42C.S522F.1         42C  S522F
## D.42C.S522F.2         42C  S522F
## D.42C.S531F.1         42C  S531F
## D.42C.S531F.2         42C  S531F
## D.42C.V146F.1         42C  V146F
## D.42C.V146F.2         42C  V146F
```

## Create DESeq object

```
# Creates DESeq object with design formula including interaction term
deseq <- DESeqDataSetFromMatrix(countData = count_table, 
                                colData = sample_table, 
                                design = ~ mutant + temperature + mutant:temperature)

# Pre-filtering to have a read count of at least 10 for a minimal number of samples 
smallestGroupSize <- 2
keep <- rowSums(counts(deseq) >= 10) >= smallestGroupSize
deseq <- deseq[keep,]

# Re-level the DESeq object so the 'reference' (default) level is WT and 37C
deseq$mutant <- relevel(deseq$mutant, ref = "WT")
deseq$temperature <- relevel(deseq$temperature, ref = "37C")
```

## Run DESeq

```
# Runs the DESeq analysis on the DESeq object made earlier
d.deseq <- DESeq(deseq)
```

```
## estimating size factors
```

```
## estimating dispersions
```

```
## gene-wise dispersion estimates
```

```
## mean-dispersion relationship
```

```
## final dispersion estimates
```

```
## fitting model and testing
```

```
# Extracts results headings from the DESeq analysis
resultsNames(d.deseq)
```

```
##  [1] "Intercept"                  "mutant_H526Y_vs_WT"        
##  [3] "mutant_Q513L_vs_WT"         "mutant_Q513R_vs_WT"        
##  [5] "mutant_S522F_vs_WT"         "mutant_S531F_vs_WT"        
##  [7] "mutant_V146F_vs_WT"         "temperature_42C_vs_37C"    
##  [9] "mutantH526Y.temperature42C" "mutantQ513L.temperature42C"
## [11] "mutantQ513R.temperature42C" "mutantS522F.temperature42C"
## [13] "mutantS531F.temperature42C" "mutantV146F.temperature42C"
```

```
# Set threshold for FDR corrected significance values
FDR_threshold <- 0.05

# Set 'Log fold change' threshold to genes that are 2x differentially expressed:
LFC_threshold <- 1

# Match gene IDs to gene names in the DEseq object
x <- read.csv("rlog_rownames.csv")
y <- read.csv("ID_to_names.csv")
join <- left_join(x, y, by = "gene_ID") %>% 
  select(-X)
deseq_filtered_genes <- as.data.frame(rownames(d.deseq)) %>% 
  rename(gene_ID = "rownames(d.deseq)") 
deseq_filtered_genes <- left_join(deseq_filtered_genes, join, by = "gene_ID") %>% 
  select(-gene_ID)
rownames(d.deseq) <- deseq_filtered_genes$gene_name
rm(x, y, join, deseq_filtered_genes)
```

## Extracting data

### Mutant vs WT (37)

```
# Ensure levels are correctly ordered so that comparisons are made in 37C

resultsNames(d.deseq)
```

```
##  [1] "Intercept"                  "mutant_H526Y_vs_WT"        
##  [3] "mutant_Q513L_vs_WT"         "mutant_Q513R_vs_WT"        
##  [5] "mutant_S522F_vs_WT"         "mutant_S531F_vs_WT"        
##  [7] "mutant_V146F_vs_WT"         "temperature_42C_vs_37C"    
##  [9] "mutantH526Y.temperature42C" "mutantQ513L.temperature42C"
## [11] "mutantQ513R.temperature42C" "mutantS522F.temperature42C"
## [13] "mutantS531F.temperature42C" "mutantV146F.temperature42C"
```

```
d.deseq$mutant <- relevel(d.deseq$mutant, "WT")
d.deseq$temperature <- relevel(d.deseq$temperature, "37C")
d.deseq <- nbinomWaldTest(d.deseq)
```

```
## found results columns, replacing these
```

```
resultsNames(d.deseq)
```

```
##  [1] "Intercept"                  "mutant_H526Y_vs_WT"        
##  [3] "mutant_Q513L_vs_WT"         "mutant_Q513R_vs_WT"        
##  [5] "mutant_S522F_vs_WT"         "mutant_S531F_vs_WT"        
##  [7] "mutant_V146F_vs_WT"         "temperature_42C_vs_37C"    
##  [9] "mutantH526Y.temperature42C" "mutantQ513L.temperature42C"
## [11] "mutantQ513R.temperature42C" "mutantS522F.temperature42C"
## [13] "mutantS531F.temperature42C" "mutantV146F.temperature42C"
```

```
# Extract separate results tables for each comparison from DESeq object

V146F_WT_37_unshrunk <- results(d.deseq, name = "mutant_V146F_vs_WT",
                         alpha = FDR_threshold,
                         lfcThreshold = LFC_threshold)
Q513L_WT_37_unshrunk <- results(d.deseq, name = "mutant_Q513L_vs_WT", 
                       alpha = FDR_threshold, 
                       lfcThreshold = LFC_threshold)
Q513R_WT_37_unshrunk <- results(d.deseq, name = "mutant_Q513R_vs_WT", 
                       alpha = FDR_threshold, 
                       lfcThreshold = LFC_threshold)
S522F_WT_37_unshrunk <- results(d.deseq, name = "mutant_S522F_vs_WT", 
                       alpha = FDR_threshold, 
                       lfcThreshold = LFC_threshold)
H526Y_WT_37_unshrunk <- results(d.deseq, name = "mutant_H526Y_vs_WT", 
                       alpha = FDR_threshold, 
                       lfcThreshold = LFC_threshold)
S531F_WT_37_unshrunk <- results(d.deseq, name = "mutant_S531F_vs_WT", 
                       alpha = FDR_threshold, 
                       lfcThreshold = LFC_threshold)

# Apply shrinkage - "Shrinkage of effect size (LFC estimates) is useful for visualization and ranking of genes"

V146F_WT_37 <- lfcShrink(d.deseq, coef = "mutant_V146F_vs_WT", res=V146F_WT_37_unshrunk, type="apeglm")
```

```
## using 'apeglm' for LFC shrinkage. If used in published research, please cite:
##     Zhu, A., Ibrahim, J.G., Love, M.I. (2018) Heavy-tailed prior distributions for
##     sequence count data: removing the noise and preserving large differences.
##     Bioinformatics. https://doi.org/10.1093/bioinformatics/bty895 [doi.org]
```

```
Q513L_WT_37 <- lfcShrink(d.deseq, coef = "mutant_Q513L_vs_WT", res=Q513L_WT_37_unshrunk, type="apeglm")
```

```
## using 'apeglm' for LFC shrinkage. If used in published research, please cite:
##     Zhu, A., Ibrahim, J.G., Love, M.I. (2018) Heavy-tailed prior distributions for
##     sequence count data: removing the noise and preserving large differences.
##     Bioinformatics. https://doi.org/10.1093/bioinformatics/bty895 [doi.org]
```

```
Q513R_WT_37 <- lfcShrink(d.deseq, coef = "mutant_Q513R_vs_WT", res=Q513R_WT_37_unshrunk, type="apeglm")
```

```
## using 'apeglm' for LFC shrinkage. If used in published research, please cite:
##     Zhu, A., Ibrahim, J.G., Love, M.I. (2018) Heavy-tailed prior distributions for
##     sequence count data: removing the noise and preserving large differences.
##     Bioinformatics. https://doi.org/10.1093/bioinformatics/bty895 [doi.org]
```

```
S522F_WT_37 <- lfcShrink(d.deseq, coef = "mutant_S522F_vs_WT", res=S522F_WT_37_unshrunk, type="apeglm")
```

```
## using 'apeglm' for LFC shrinkage. If used in published research, please cite:
##     Zhu, A., Ibrahim, J.G., Love, M.I. (2018) Heavy-tailed prior distributions for
##     sequence count data: removing the noise and preserving large differences.
##     Bioinformatics. https://doi.org/10.1093/bioinformatics/bty895 [doi.org]
```

```
H526Y_WT_37 <- lfcShrink(d.deseq, coef = "mutant_H526Y_vs_WT", res=H526Y_WT_37_unshrunk, type="apeglm")
```

```
## using 'apeglm' for LFC shrinkage. If used in published research, please cite:
##     Zhu, A., Ibrahim, J.G., Love, M.I. (2018) Heavy-tailed prior distributions for
##     sequence count data: removing the noise and preserving large differences.
##     Bioinformatics. https://doi.org/10.1093/bioinformatics/bty895 [doi.org]
```

```
S531F_WT_37 <- lfcShrink(d.deseq, coef = "mutant_S531F_vs_WT", res=S531F_WT_37_unshrunk, type="apeglm")
```

```
## using 'apeglm' for LFC shrinkage. If used in published research, please cite:
##     Zhu, A., Ibrahim, J.G., Love, M.I. (2018) Heavy-tailed prior distributions for
##     sequence count data: removing the noise and preserving large differences.
##     Bioinformatics. https://doi.org/10.1093/bioinformatics/bty895 [doi.org]
```

```
# Save significantly differentially expressed gene list to data frame

sigV146F_WT_37 <- as.data.frame(subset(V146F_WT_37, padj < FDR_threshold & abs(log2FoldChange) > LFC_threshold)) %>% tibble::rownames_to_column("genes")
sigQ513L_WT_37 <- as.data.frame(subset(Q513L_WT_37, padj < FDR_threshold & abs(log2FoldChange) > LFC_threshold)) %>% tibble::rownames_to_column("genes")
sigQ513R_WT_37 <- as.data.frame(subset(Q513R_WT_37, padj < FDR_threshold & abs(log2FoldChange) > LFC_threshold)) %>% tibble::rownames_to_column("genes")
sigS522F_WT_37 <- as.data.frame(subset(S522F_WT_37, padj < FDR_threshold & abs(log2FoldChange) > LFC_threshold)) %>% tibble::rownames_to_column("genes")
sigH526Y_WT_37 <- as.data.frame(subset(H526Y_WT_37, padj < FDR_threshold & abs(log2FoldChange) > LFC_threshold)) %>% tibble::rownames_to_column("genes")
sigS531F_WT_37 <- as.data.frame(subset(S531F_WT_37, padj < FDR_threshold & abs(log2FoldChange) > LFC_threshold)) %>% tibble::rownames_to_column("genes")

write.csv(sigV146F_WT_37, "sig V146F vs WT 37.csv")
write.csv(sigQ513L_WT_37, "sig Q513L vs WT 37.csv")
write.csv(sigQ513R_WT_37, "sig Q513R vs WT 37.csv")
write.csv(sigS522F_WT_37, "sig S522F vs WT 37.csv")
write.csv(sigH526Y_WT_37, "sig H526Y vs WT 37.csv")
write.csv(sigS531F_WT_37, "sig S531F vs WT 37.csv")

rm(V146F_WT_37_unshrunk, Q513L_WT_37_unshrunk, Q513R_WT_37_unshrunk, 
   S522F_WT_37_unshrunk, H526Y_WT_37_unshrunk, S531F_WT_37_unshrunk,
   V146F_WT_37, Q513L_WT_37, Q513R_WT_37, S522F_WT_37, H526Y_WT_37, S531F_WT_37)
```

### Mutant vs WT (42)

```
# Re-level DESeq object so that comparisons are made in 42C

resultsNames(d.deseq)
```

```
##  [1] "Intercept"                  "mutant_H526Y_vs_WT"        
##  [3] "mutant_Q513L_vs_WT"         "mutant_Q513R_vs_WT"        
##  [5] "mutant_S522F_vs_WT"         "mutant_S531F_vs_WT"        
##  [7] "mutant_V146F_vs_WT"         "temperature_42C_vs_37C"    
##  [9] "mutantH526Y.temperature42C" "mutantQ513L.temperature42C"
## [11] "mutantQ513R.temperature42C" "mutantS522F.temperature42C"
## [13] "mutantS531F.temperature42C" "mutantV146F.temperature42C"
```

```
d.deseq$temperature <- relevel(d.deseq$temperature, "42C")
d.deseq <- nbinomWaldTest(d.deseq)
```

```
## found results columns, replacing these
```

```
resultsNames(d.deseq)
```

```
##  [1] "Intercept"                  "mutant_H526Y_vs_WT"        
##  [3] "mutant_Q513L_vs_WT"         "mutant_Q513R_vs_WT"        
##  [5] "mutant_S522F_vs_WT"         "mutant_S531F_vs_WT"        
##  [7] "mutant_V146F_vs_WT"         "temperature_37C_vs_42C"    
##  [9] "mutantH526Y.temperature37C" "mutantQ513L.temperature37C"
## [11] "mutantQ513R.temperature37C" "mutantS522F.temperature37C"
## [13] "mutantS531F.temperature37C" "mutantV146F.temperature37C"
```

```
# Extract seperate results tables from DESeq object (d.deseq)

V146F_WT_42_unshrunk <- results(d.deseq, name = "mutant_V146F_vs_WT",
                                alpha = FDR_threshold,
                                lfcThreshold = LFC_threshold)
Q513L_WT_42_unshrunk <- results(d.deseq, name = "mutant_Q513L_vs_WT", 
                                alpha = FDR_threshold, 
                                lfcThreshold = LFC_threshold)
Q513R_WT_42_unshrunk <- results(d.deseq, name = "mutant_Q513R_vs_WT", 
                                alpha = FDR_threshold,
                                lfcThreshold = LFC_threshold)
S522F_WT_42_unshrunk <- results(d.deseq, name = "mutant_S522F_vs_WT", 
                                alpha = FDR_threshold, 
                                lfcThreshold = LFC_threshold)
H526Y_WT_42_unshrunk <- results(d.deseq, name = "mutant_H526Y_vs_WT", 
                                alpha = FDR_threshold, 
                                lfcThreshold = LFC_threshold)
S531F_WT_42_unshrunk <- results(d.deseq, name = "mutant_S531F_vs_WT", 
                                alpha = FDR_threshold, 
                                lfcThreshold = LFC_threshold)

# Apply shrinkage
# Shrinkage of effect size (LFC estimates) is useful for visualization and ranking of genes

V146F_WT_42 <- lfcShrink(d.deseq, coef = "mutant_V146F_vs_WT", res=V146F_WT_42_unshrunk, type="apeglm")
```

```
## using 'apeglm' for LFC shrinkage. If used in published research, please cite:
##     Zhu, A., Ibrahim, J.G., Love, M.I. (2018) Heavy-tailed prior distributions for
##     sequence count data: removing the noise and preserving large differences.
##     Bioinformatics. https://doi.org/10.1093/bioinformatics/bty895 [doi.org]
```

```
Q513L_WT_42 <- lfcShrink(d.deseq, coef = "mutant_Q513L_vs_WT", res=Q513L_WT_42_unshrunk, type="apeglm")
```

```
## using 'apeglm' for LFC shrinkage. If used in published research, please cite:
##     Zhu, A., Ibrahim, J.G., Love, M.I. (2018) Heavy-tailed prior distributions for
##     sequence count data: removing the noise and preserving large differences.
##     Bioinformatics. https://doi.org/10.1093/bioinformatics/bty895 [doi.org]
```

```
Q513R_WT_42 <- lfcShrink(d.deseq, coef = "mutant_Q513R_vs_WT", res=Q513R_WT_42_unshrunk, type="apeglm")
```

```
## using 'apeglm' for LFC shrinkage. If used in published research, please cite:
##     Zhu, A., Ibrahim, J.G., Love, M.I. (2018) Heavy-tailed prior distributions for
##     sequence count data: removing the noise and preserving large differences.
##     Bioinformatics. https://doi.org/10.1093/bioinformatics/bty895 [doi.org]
```

```
S522F_WT_42 <- lfcShrink(d.deseq, coef = "mutant_S522F_vs_WT", res=S522F_WT_42_unshrunk, type="apeglm")
```

```
## using 'apeglm' for LFC shrinkage. If used in published research, please cite:
##     Zhu, A., Ibrahim, J.G., Love, M.I. (2018) Heavy-tailed prior distributions for
##     sequence count data: removing the noise and preserving large differences.
##     Bioinformatics. https://doi.org/10.1093/bioinformatics/bty895 [doi.org]
```

```
H526Y_WT_42 <- lfcShrink(d.deseq, coef = "mutant_H526Y_vs_WT", res=H526Y_WT_42_unshrunk, type="apeglm")
```

```
## using 'apeglm' for LFC shrinkage. If used in published research, please cite:
##     Zhu, A., Ibrahim, J.G., Love, M.I. (2018) Heavy-tailed prior distributions for
##     sequence count data: removing the noise and preserving large differences.
##     Bioinformatics. https://doi.org/10.1093/bioinformatics/bty895 [doi.org]
```

```
S531F_WT_42 <- lfcShrink(d.deseq, coef = "mutant_S531F_vs_WT", res=S531F_WT_42_unshrunk, type="apeglm")
```

```
## using 'apeglm' for LFC shrinkage. If used in published research, please cite:
##     Zhu, A., Ibrahim, J.G., Love, M.I. (2018) Heavy-tailed prior distributions for
##     sequence count data: removing the noise and preserving large differences.
##     Bioinformatics. https://doi.org/10.1093/bioinformatics/bty895 [doi.org]
```

```
# Save differentially expressed gene list to data frame

sigV146F_WT_42 <- as.data.frame(subset(V146F_WT_42, padj < FDR_threshold & abs(log2FoldChange) > LFC_threshold)) %>% tibble::rownames_to_column("genes")
sigQ513L_WT_42 <- as.data.frame(subset(Q513L_WT_42, padj < FDR_threshold & abs(log2FoldChange) > LFC_threshold)) %>% tibble::rownames_to_column("genes")
sigQ513R_WT_42 <- as.data.frame(subset(Q513R_WT_42, padj < FDR_threshold & abs(log2FoldChange) > LFC_threshold)) %>% tibble::rownames_to_column("genes")
sigS522F_WT_42 <- as.data.frame(subset(S522F_WT_42, padj < FDR_threshold & abs(log2FoldChange) > LFC_threshold)) %>% tibble::rownames_to_column("genes")
sigH526Y_WT_42 <- as.data.frame(subset(H526Y_WT_42, padj < FDR_threshold & abs(log2FoldChange) > LFC_threshold)) %>% tibble::rownames_to_column("genes")
sigS531F_WT_42 <- as.data.frame(subset(S531F_WT_42, padj < FDR_threshold & abs(log2FoldChange) > LFC_threshold)) %>% tibble::rownames_to_column("genes")


write.csv(sigV146F_WT_42, "sig V146F vs WT 42.csv")
write.csv(sigQ513L_WT_42, "sig Q513L vs WT 42.csv")
write.csv(sigQ513R_WT_42, "sig Q513R vs WT 42.csv")
write.csv(sigS522F_WT_42, "sig S522F vs WT 42.csv")
write.csv(sigH526Y_WT_42, "sig H526Y vs WT 42.csv")
write.csv(sigS531F_WT_42, "sig S531F vs WT 42.csv")


rm(V146F_WT_42_unshrunk, Q513L_WT_42_unshrunk, Q513R_WT_42_unshrunk, 
   S522F_WT_42_unshrunk, H526Y_WT_42_unshrunk, S531F_WT_42_unshrunk,
   V146F_WT_42, Q513L_WT_42, Q513R_WT_42, S522F_WT_42, H526Y_WT_42, S531F_WT_42)
```

### 42 vs 37

```
# For this comparison (the 'condition effect'), the required coefficient in resultsNames(d.deseq) is "temperature_42C_vs_37C". Here we explicitly relevel the mutant variable for each comparison. The initial one will be WT 42 vs WT 37, since WT was initially set as the reference level for mutant.

resultsNames(d.deseq)
```

```
##  [1] "Intercept"                  "mutant_H526Y_vs_WT"        
##  [3] "mutant_Q513L_vs_WT"         "mutant_Q513R_vs_WT"        
##  [5] "mutant_S522F_vs_WT"         "mutant_S531F_vs_WT"        
##  [7] "mutant_V146F_vs_WT"         "temperature_37C_vs_42C"    
##  [9] "mutantH526Y.temperature37C" "mutantQ513L.temperature37C"
## [11] "mutantQ513R.temperature37C" "mutantS522F.temperature37C"
## [13] "mutantS531F.temperature37C" "mutantV146F.temperature37C"
```

```
d.deseq$temperature <- relevel(d.deseq$temperature, "37C")
d.deseq <- nbinomWaldTest(d.deseq)
```

```
## found results columns, replacing these
```

```
resultsNames(d.deseq)
```

```
##  [1] "Intercept"                  "mutant_H526Y_vs_WT"        
##  [3] "mutant_Q513L_vs_WT"         "mutant_Q513R_vs_WT"        
##  [5] "mutant_S522F_vs_WT"         "mutant_S531F_vs_WT"        
##  [7] "mutant_V146F_vs_WT"         "temperature_42C_vs_37C"    
##  [9] "mutantH526Y.temperature42C" "mutantQ513L.temperature42C"
## [11] "mutantQ513R.temperature42C" "mutantS522F.temperature42C"
## [13] "mutantS531F.temperature42C" "mutantV146F.temperature42C"
```

```
mutants <- c("WT", "V146F", "Q513L", "Q513R", "S522F", "H526Y", "S531F")

for (mutant in mutants) {
  # Relevel 
  d.deseq$mutant <- relevel(d.deseq$mutant, mutant)
  d.deseq <- nbinomWaldTest(d.deseq)
  resultsNames(d.deseq)
  
  # Extract comparison
  `42_vs_37_unshrunk` <- results(d.deseq, name = "temperature_42C_vs_37C", 
                             alpha = FDR_threshold, 
                             lfcThreshold = LFC_threshold)
  
  # Shrink data
  `42_vs_37` <- lfcShrink(d.deseq, coef = "temperature_42C_vs_37C", res=`42_vs_37_unshrunk`, type="apeglm")
  assign(paste0(mutant, "_42_vs_37"), `42_vs_37`)
  
  # Pull significant gene list
  sig_42_vs_37 <- as.data.frame(subset(`42_vs_37`, padj < FDR_threshold & abs(log2FoldChange) > LFC_threshold))
  
  # Export to CSV
  write.csv(sig_42_vs_37, paste0(mutant, " 42C vs 37C.csv"))

}
```

```
## found results columns, replacing these
```

```
## using 'apeglm' for LFC shrinkage. If used in published research, please cite:
##     Zhu, A., Ibrahim, J.G., Love, M.I. (2018) Heavy-tailed prior distributions for
##     sequence count data: removing the noise and preserving large differences.
##     Bioinformatics. https://doi.org/10.1093/bioinformatics/bty895 [doi.org]
```

```
## found results columns, replacing these
```

```
## using 'apeglm' for LFC shrinkage. If used in published research, please cite:
##     Zhu, A., Ibrahim, J.G., Love, M.I. (2018) Heavy-tailed prior distributions for
##     sequence count data: removing the noise and preserving large differences.
##     Bioinformatics. https://doi.org/10.1093/bioinformatics/bty895 [doi.org]
```

```
## some rows did not converge in finding the MAP
```

```
## found results columns, replacing these
```

```
## using 'apeglm' for LFC shrinkage. If used in published research, please cite:
##     Zhu, A., Ibrahim, J.G., Love, M.I. (2018) Heavy-tailed prior distributions for
##     sequence count data: removing the noise and preserving large differences.
##     Bioinformatics. https://doi.org/10.1093/bioinformatics/bty895 [doi.org]
```

```
## found results columns, replacing these
```

```
## using 'apeglm' for LFC shrinkage. If used in published research, please cite:
##     Zhu, A., Ibrahim, J.G., Love, M.I. (2018) Heavy-tailed prior distributions for
##     sequence count data: removing the noise and preserving large differences.
##     Bioinformatics. https://doi.org/10.1093/bioinformatics/bty895 [doi.org]
```

```
## found results columns, replacing these
```

```
## using 'apeglm' for LFC shrinkage. If used in published research, please cite:
##     Zhu, A., Ibrahim, J.G., Love, M.I. (2018) Heavy-tailed prior distributions for
##     sequence count data: removing the noise and preserving large differences.
##     Bioinformatics. https://doi.org/10.1093/bioinformatics/bty895 [doi.org]
```

```
## some rows did not converge in finding the MAP
```

```
## found results columns, replacing these
```

```
## using 'apeglm' for LFC shrinkage. If used in published research, please cite:
##     Zhu, A., Ibrahim, J.G., Love, M.I. (2018) Heavy-tailed prior distributions for
##     sequence count data: removing the noise and preserving large differences.
##     Bioinformatics. https://doi.org/10.1093/bioinformatics/bty895 [doi.org]
```

```
## found results columns, replacing these
```

```
## using 'apeglm' for LFC shrinkage. If used in published research, please cite:
##     Zhu, A., Ibrahim, J.G., Love, M.I. (2018) Heavy-tailed prior distributions for
##     sequence count data: removing the noise and preserving large differences.
##     Bioinformatics. https://doi.org/10.1093/bioinformatics/bty895 [doi.org]
```

```
## some rows did not converge in finding the MAP
```

```
# Reading back in each dataframe

sig_WT_vs_WT <- read.csv("WT 42C vs 37C.csv") %>% rename("gene" = X)
sig_V146F_vs_V146F <- read.csv("V146F 42C vs 37C.csv") %>% rename("gene" = X)
sig_Q513L_vs_Q513L <- read.csv("Q513L 42C vs 37C.csv") %>% rename("gene" = X)
sig_Q513R_vs_Q513R <- read.csv("Q513R 42C vs 37C.csv") %>% rename("gene" = X)
sig_S522F_vs_S522F <- read.csv("S522F 42C vs 37C.csv") %>% rename("gene" = X)
sig_H526Y_vs_H526Y <- read.csv("H526Y 42C vs 37C.csv") %>% rename("gene" = X)
sig_S531F_vs_S531F <- read.csv("S531F 42C vs 37C.csv") %>% rename("gene" = X)
```

### Re-read data tables

```
sigV146F_WT_37 <- read.csv("sig V146F vs WT 37.csv")
sigQ513L_WT_37 <- read.csv("sig Q513L vs WT 37.csv")
sigQ513R_WT_37 <- read.csv("sig Q513R vs WT 37.csv")
sigS522F_WT_37 <- read.csv("sig S522F vs WT 37.csv")
sigH526Y_WT_37 <- read.csv("sig H526Y vs WT 37.csv")
sigS531F_WT_37 <- read.csv("sig S531F vs WT 37.csv")
  
sigV146F_WT_42 <- read.csv("sig V146F vs WT 42.csv")
sigQ513L_WT_42 <- read.csv("sig Q513L vs WT 42.csv")
sigQ513R_WT_42 <- read.csv("sig Q513R vs WT 42.csv")
sigS522F_WT_42 <- read.csv("sig S522F vs WT 42.csv")
sigH526Y_WT_42 <- read.csv("sig H526Y vs WT 42.csv")
sigS531F_WT_42 <- read.csv("sig S531F vs WT 42.csv")

sig_WT_vs_WT <- read.csv("WT 42C vs 37C.csv")
sig_V146F_vs_V146F <- read.csv("V146F 42C vs 37C.csv")
sig_Q513L_vs_Q513L <- read.csv("Q513L 42C vs 37C.csv")
sig_Q513R_vs_Q513R <- read.csv("Q513R 42C vs 37C.csv")
sig_S522F_vs_S522F <- read.csv("S522F 42C vs 37C.csv")
sig_H526Y_vs_H526Y <- read.csv("H526Y 42C vs 37C.csv")
sig_S531F_vs_S531F <- read.csv("S531F 42C vs 37C.csv")
```

## DEG bar plots

```
palette <- c("#B71C1C", "#1565C0")

## Mutant vs WT 37C

# Merge data into one dataframe
mutant_vs_WT_37 <- bind_rows("V146F" = sigV146F_WT_37, 
                             "Q513L" = sigQ513L_WT_37, 
                             "Q513R" = sigQ513R_WT_37,
                             "S522F" = sigS522F_WT_37,
                             "H526Y" = sigH526Y_WT_37,
                             "S531F" = sigS531F_WT_37,
                             .id = "mutant")

# Classify genes as up- or downregulated
mutant_vs_WT_37 <- mutant_vs_WT_37 %>%
  select(mutant, 2, log2FoldChange, padj) %>%
  mutate(direction = case_when(log2FoldChange > 0 ~ "UP", log2FoldChange < 0 ~ "DOWN")) 

# Reorder mutants so they appear in plots in correct order
mutant_vs_WT_37 <- mutant_vs_WT_37 %>% 
  mutate(mutant = factor(mutant, levels = rev(mutants)))

# Make bar plot
pmutant_vs_WT_37 <- mutant_vs_WT_37 %>% ggplot(aes(x = mutant, fill = direction)) +
  geom_bar(position = position_dodge(preserve = "single")) + 
  ggtitle("Mutant vs WT (37C)") +
  xlab(element_blank()) + 
  ylab("Number of genes") + 
  labs(fill = "Direction") + 
  guides(fill = guide_legend(reverse=TRUE)) +
  theme_minimal() + 
  coord_flip() + 
  scale_y_reverse(limits = c(200,0)) + 
  scale_x_discrete(position = "top") + 
  scale_fill_manual(values = rev(palette)) 


## Mutant vs WT 42C

# Merge data into one dataframe
mutant_vs_WT_42 <- bind_rows("V146F" = sigV146F_WT_42, 
                             "Q513L" = sigQ513L_WT_42, 
                             "Q513R" = sigQ513R_WT_42,
                             "S522F" = sigS522F_WT_42,
                             "H526Y" = sigH526Y_WT_42,
                             "S531F" = sigS531F_WT_42,
                             .id = "mutant")

# Classify genes as up- or downregulated
mutant_vs_WT_42 <- mutant_vs_WT_42 %>%
  select(mutant, 2, log2FoldChange, padj) %>%
  mutate(direction = case_when(log2FoldChange > 0 ~ "UP", log2FoldChange < 0 ~ "DOWN")) 

# Reorder mutants so they appear in plots in correct order
mutant_vs_WT_42 <- mutant_vs_WT_42 %>% 
  mutate(mutant = factor(mutant, levels = rev(mutants)))

# Make bar plot 
pmutant_vs_WT_42 <- mutant_vs_WT_42 %>% ggplot(aes(x = mutant, fill = direction)) +
  geom_bar(position = position_dodge(preserve = "single")) + 
  ggtitle("Mutant vs WT (42C)") +
  xlab(element_blank()) + 
  ylab("Number of genes") + 
  labs(fill = "Direction") + 
  guides(fill = guide_legend(reverse=TRUE)) +
  coord_flip() + 
  scale_fill_manual(values = rev(palette)) +
  ylim(0, 200) +
  theme_minimal() + 
  theme(axis.text.y = element_blank()) 

## 42C vs 37C

all_42_vs_37 <- bind_rows("WT" = sig_WT_vs_WT,
                          "V146F" = sig_V146F_vs_V146F,
                          "Q513L" = sig_Q513L_vs_Q513L,
                          "Q513R" = sig_Q513R_vs_Q513R,
                          "S522F" = sig_S522F_vs_S522F,
                          "H526Y" = sig_H526Y_vs_H526Y,
                          "S531F" = sig_S531F_vs_S531F,
                          .id = "mutant")

# Classify genes as up- or downregulated
all_42_vs_37 <- all_42_vs_37 %>%
  select(mutant, 2, log2FoldChange, padj) %>%
  mutate(direction = case_when(log2FoldChange > 0 ~ "UP", log2FoldChange < 0 ~ "DOWN")) 

# Reorder mutants so they appear in plots in correct order
all_42_vs_37 <- all_42_vs_37 %>% 
  mutate(mutant = factor(mutant, levels = mutants))

# Make bar plot 
p42C_vs_37C <- all_42_vs_37 %>% ggplot(aes(x = mutant, fill = direction)) +
  geom_bar(position = position_dodge(preserve = "single")) + 
  ggtitle("42C vs 37C") +
  xlab("Mutant") + 
  ylab("Number of genes") + 
  labs(fill = "Direction") + 
  guides(fill = guide_legend(reverse=TRUE)) +
  ylim(0, 250) + 
  scale_fill_manual(values = rev(palette)) +
  theme_bw()


# Paste both plots together
((pmutant_vs_WT_37 + pmutant_vs_WT_42) / p42C_vs_37C) + patchwork::plot_layout(guides = 'collect')
```
