# Supplementary material for: Deciphering the Impact of Temperature on Pleiotropic Consequences of RNA Polymerase Mutations
Source: Mol Biol Evol. 2025 Sep 16;42(10):msaf226. doi: 10.1093/molbev/msaf226 (PMC12503110; doi:10.1093/molbev/msaf226)
Supplement: msaf226_Supplementary_Data [file msaf226_supplementary_data.zip › Supplementary_tables_and_figures_.pdf]

Supplementary Table 1. All RNA polymerase PDB structures used in the study. We used multiple PDB structures to capture a range of structural conformations of RNAP bound to different σ-factors and different stages of transcription

PDB ID	Structure notes	Where data is used	Reference
4KMU	*In complex with rifampicin*	*Figure 2A*<br>*Supplementary figure 1*	[56]
6UU9	*σ -S, initiation complex, 8-nt nascent RNA*	*Figure 2B*<br>*Figure 2C (Template/non-template DNA, RNA, β', σ factor, ΔΔG)*<br>*Figure 4E*<br>*Supplementary figure 1*	[57]
6PMJ	*σ -28, initiation complex, 3-nt nascent RNA*	*Figure 2C (Template/non-template DNA, β', σ factor, ΔΔG)*<br>*Figure 4D,E*<br>*Supplementary figure 1*	[58]
8HKC	*σ -32, initiation complex*	*Figure 2C (Template/non-template DNA, β', σ factor, ΔΔG)*<br>*Supplementary figure 1*	[59]
5IPL	*σ -S, initiation complex, 4-nt nascent RNA*	*Figure 2C (Template/non-template DNA, RNA, β', σ factor, ΔΔG)*<br>*Figure 4D,E*<br>*Supplementary figure 1*	[60]
6GFW	*σ -54, initiation complex, 4-nt nascent RNA*	*Figure 2C (Template/non-template DNA, β', σ factor, ΔΔG)*<br>*Supplementary figure 1*	[61]
6JBQ	*σ -24, initiation complex, 5-nt nascent RNA*	*Figure 2C (Template/non-template DNA, β', σ factor, ΔΔG)*<br>*Supplementary figure 1*	[62]
6N62	*σ -70*	*Figure 2C (β', σ factor ΔΔG)*<br>*Supplementary figure 1*	[63]
7MKP	*RNAP core enzyme*	*Figure 2C (ΔΔG)*<br>*Supplementary figure 1*	[64]
6ALH	*Elongation complex*	*Figure 2C (ΔΔG)*	[65]
6C9Y	*σ -70*	*Figure 2C (ΔΔG)*<br>*Supplementary figure 1*	[66]

Supplementary Table 2. Permutational multivariate analysis of variance (PERMANOVA) model fitted to the normalised RNAseq count data matrix.

Effect	Degrees of freedom	Sum of squares	R^2	F-statistic	Pr(>F)
Temperature	1	0.40	0.17	15.5	0.0001
Mutation	6	0.69	0.30	4.43	0.0001
Experiment	1	0.56	0.02	2.14	0.0594
Replicate	3	0.16	0.07	2.06	0.0147
Temperature:Mutation	5	0.67	0.29	5.16	0.0001
Residual	13	0.34	0.15	NA	NA
Total	29	2.30	1.00	NA	NA

Supplementary Table 3. Numbers of differentially expressed genes shared between genotypes at the 42°C vs. 37°C.

	WT	V146F	Q513L	Q513R	S531F	S522F
H526Y	60	29	14	24	45	76
S522F	43	109	61	28	64	
S531F	44	37	48	56		
Q513R	51	15	17			
Q513L	28	55				
V146F	28					

Supplementary Table 4. Statistical outputs of all correlations carried out in the study. Provided as excel spreadsheet.

Supplementary Table 5. Output of t-test performed on areas under the curve (AUC) estimated using the *growthcurver* package obtained after 24 hours of growth of the six RNA polymerase mutants and the wildtype at 37°C and 42°C. Six biological replicates (n = 6) tested for all RNA polymerase mutants and the wildtype.

sample	p value	t statistic	confidence interval-lower	confidence interval-upper
H526Y	0.120	-1.65	-4.27	0.550
Q513L	0.190	-1.39	-3.20	0.700
Q513R	3.3E-07	-9.62	-8.39	-5.31
S522F	0.560	0.600	-2.62	1.45
S531F	0.00500	-3.41	-5.85	-1.29
V146F	0.0510	-2.13	-3.63	0.00900
WT	0.150	-1.54	-3.08	0.510

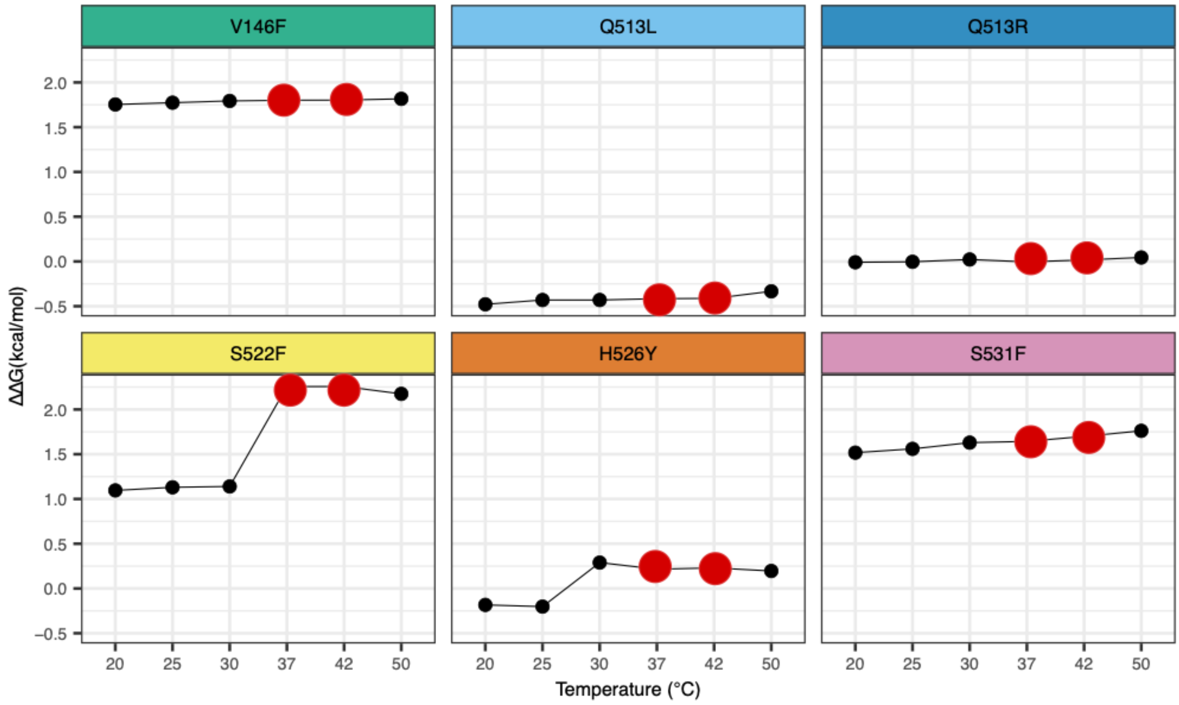

Supplementary Figure 1.Temperature effect on folding stability. ΔΔG values of the six rpoB mutations calculated at six different temperatures using FoldX 'temperature' parameter using all structures examined in this study (PDB IDs can be found in Supplementary Table 1). Each point is the average ΔΔG across all tested structures. Bigger red points indicate temperatures of interest, 37°C and 42°C.

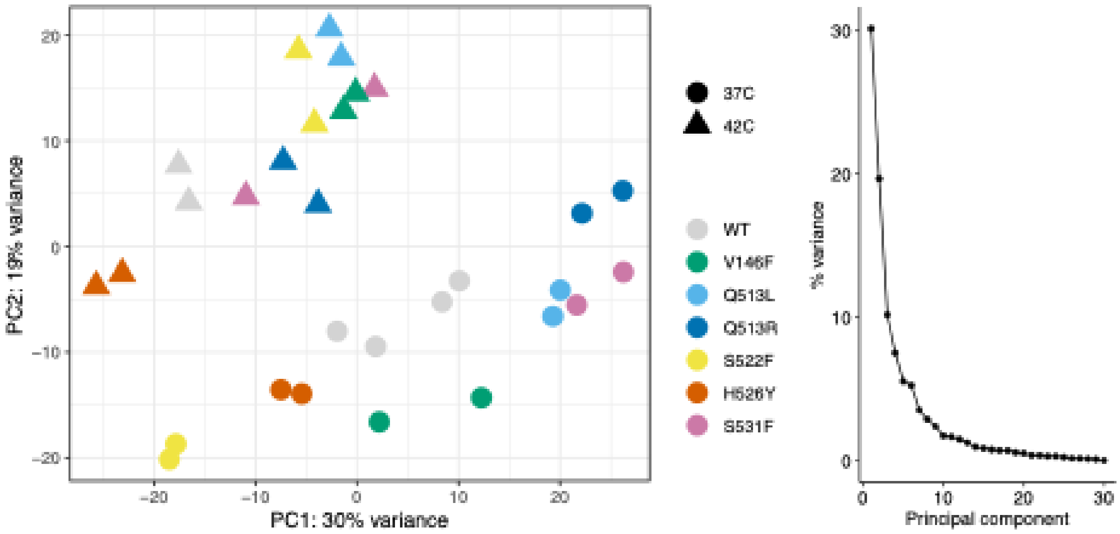

Supplementary Figure 2. Principal component analysis (PCA) of regularised log transformed normalised counts, showing Principal Component 1 vs 2 (left) that differentiate by genotype and temperature; scree plot showing percent variance of each principal component (right).

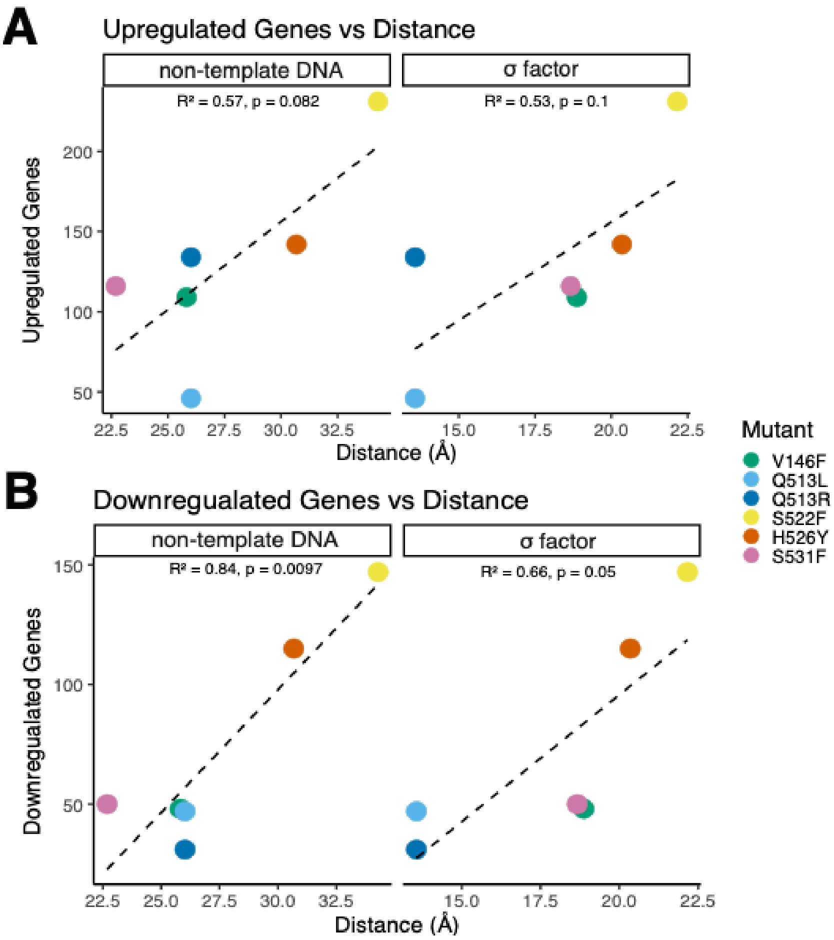

Supplementary Figure 3. Relationship between distance from resistance mutation and number of differentially expressed genes. Scatter plots show the correlation between the number of (A) upregulated and (B) downregulated genes and the structural distance (in Å) from the resistance mutation to either the non-template DNA strand (left panels) or the σ factor (right panels). Each point represents the mean distance across 11 RNAP structures (see Supplementary Table 1 for full list). Dashed lines indicate linear regression fits.

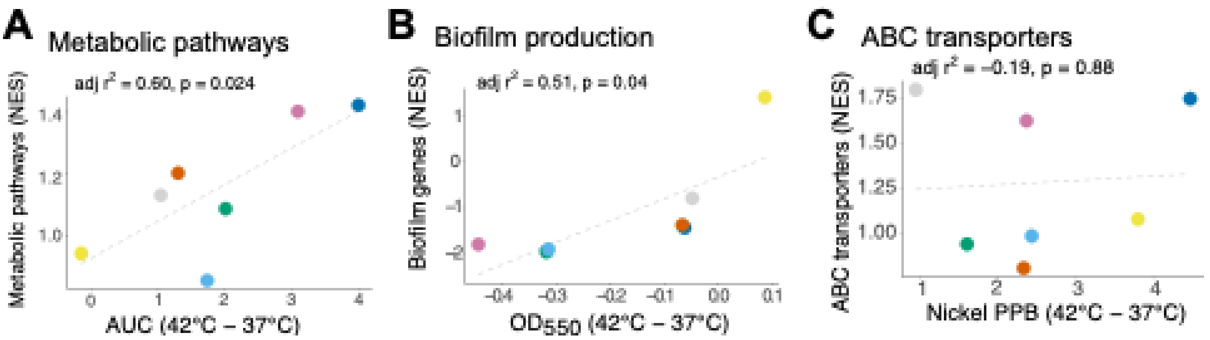

Supplementary Figure 4. Correlation between measured organismal level effects and NES of the associated functional category. A) NES for 'Metabolic pathways' KEGG group for each genotype vs. area under the curve (AUC). (B) NES for 'Biofilm formation' KEGG group for each genotype vs. difference in level of biofilm. (C) NES for 'ABC transporters' KEGG group for each genotype vs. cellular nickel parts per billion. Note that the NES and the relevant measured organismal level effects are not significant for most mutants (see Figure 6B).
